# The C-Terminal Domain of the Bacterial SSB Protein Acts as a DNA Maintenance Hub at Active Chromosome Replication Forks

**DOI:** 10.1371/journal.pgen.1001238

**Published:** 2010-12-09

**Authors:** Audrey Costes, François Lecointe, Stephen McGovern, Sophie Quevillon-Cheruel, Patrice Polard

**Affiliations:** 1 Laboratoire de Microbiologie et Génétique Moléculaires, Université de Toulouse, Centre National de la Recherche Scientifique, LMGM-UMR5100, Toulouse, France; 2 INRA, UMR1319 Micalis (Microbiologie de l'Alimentation au service de la Santé), Domaine de Vilvert, Jouy-en-Josas, France; 3 Institut de Biochimie et de Biophysique Moléculaire et Cellulaire, Université de Paris-Sud, Centre National de la Recherche Scientifique, UMR8619, IFR115, Orsay, France; Université Paris Descartes, INSERM U571, France

## Abstract

We have investigated *in vivo* the role of the carboxy-terminal domain of the *Bacillus subtilis* Single-Stranded DNA Binding protein (SSB_Cter_) as a recruitment platform at active chromosomal forks for many proteins of the genome maintenance machineries. We probed this SSB_Cter_ interactome using GFP fusions and by Tap-tag and biochemical analysis. It includes at least 12 proteins. The interactome was previously shown to include PriA, RecG, and RecQ and extended in this study by addition of DnaE, SbcC, RarA, RecJ, RecO, XseA, Ung, YpbB, and YrrC. Targeting of YpbB to active forks appears to depend on RecS, a RecQ paralogue, with which it forms a stable complex. Most of these SSB partners are conserved in bacteria, while others, such as the essential DNA polymerase DnaE, YrrC, and the YpbB/RecS complex, appear to be specific to *B. subtilis*. SSB_Cter_ deletion has a moderate impact on *B. subtilis* cell growth. However, it markedly affects the efficiency of repair of damaged genomic DNA and arrested replication forks. *ssbΔCter* mutant cells appear deficient in RecA loading on ssDNA, explaining their inefficiency in triggering the SOS response upon exposure to genotoxic agents. Together, our findings show that the bacterial SSB_Cter_ acts as a DNA maintenance hub at active chromosomal forks that secures their propagation along the genome.

## Author Summary

Cell multiplication relies primarily on the complete and accurate duplication of the genome. Thus, all organisms have evolved multiple mechanisms to protect, repair, and re-activate the DNA replication forks. A large body of research is currently aimed at deciphering the mechanisms that precisely direct the proteins involved in these rescue pathways towards the chromosome replication forks. Here, we have used the model bacterium *Bacillus subtilis* to demonstrate that the active chromosomal DNA replication forks are pre-equipped with many such rescue effectors *via* their direct physical interaction with the carboxy-terminal end (Cter) of the Single-Stranded DNA Binding protein (SSB). A detailed analysis of the multiple defects of viable *B. subtilis* mutants deleted for the Cter of SSB (SSB_Cter_) revealed the vital role of this domain for the maintenance of genome integrity and fork propagation. The inability to grow at high temperature is a major defect of the *ssbΔCter* mutant. We show that this lethality can be specifically suppressed by overexpression of RecO, one of the numerous partners of SSB, apparently by mediating the loading of the RecA recombinase on ssDNA.

## Introduction

Maintaining genome integrity is a permanent challenge for all organisms, particularly during genome duplication, when accidental replication fork arrests expose the genome to damage. Numerous mechanisms have evolved to counteract the deleterious consequences of fork arrest (reviewed in [1], [2]). The multiplicity of these fork repair mechanisms reflects the need to respond appropriately to a variety of damaged fork structures. A key question is therefore how these multiple rescue pathways are appropriately and efficiently triggered and coordinated in the cell.

Bacteria can manage chromosomal replication fork arrest without necessarily interrupting other key cell cycle events. Their genome is generally composed of one circular DNA molecule (of several Mbp) replicated by a single pair of divergent forks fired at a fixed origin, *ori*C. Thus, effective repair of accidentally arrested replication forks is vital to bacteria. In addition to a requirement for removal and repair of the damage originally responsible for a particular replication fork arrest, the cell possesses the machinery necessary for re-assembling the replication machinery (the replisome) at these rescued forks [3]. An emerging model is that components of the replisome determine the recruitment of accessory proteins at the forks to assist their progression. One of these is DnaN, a dimeric protein that forms a ring around double-stranded DNA (dsDNA) and clamps the replicative DNA polymerase [4], and also interacts with several proteins involved in DNA replication and repair (reviewed in [5]). Another protein of the replisome, the Single-Stranded DNA Binding protein (SSB), is also known to interact with accessory proteins at the fork. The primary role of SSB at the fork is to facilitate the activities of replisomal enzymes by preventing the formation of ssDNA secondary structures (for a review, see [6]). SSB is composed of two domains: an N-terminal ssDNA binding domain and a C-terminal domain, SSB_Cter_, enriched in glycine and acidic amino-acids. A short hexapeptide motif with a consensus signature D-D-D-I/L-P-F emerges from the end of the protein [7]. The SSB_Cter_ is dispensable for SSB tetramerisation and interaction with ssDNA [8], [9] but permits interaction with many proteins of the DNA recombination, repair and replication machineries. The *E. coli* SSB (*_Ec_*SSB) interactome is currently estimated to include 14 proteins (reviewed in [6]).

Many of the SSB partners are involved in distinct replication fork repair pathways. Thus, SSB might be responsible for coordinating recruitment of these repair proteins at active replication forks. As judged by the analysis of SSB localization in *B. subtilis* and *E.* coli [10]–[12], active forks are the subcellular sites where SSB accumulates in replicating cells grown without genotoxic stress. We previously provided strong support for the idea that SSB acts as a protein recruitment platform at active replication forks by localizing in living *B. subtilis* cells three conserved DNA helicases as GFP fusions. These were PriA, the primary restart protein, which directs replisome re-assembly on branched DNA originating from arrested forks [13]–[15], and RecG and RecQ, two recombination proteins involved in the maintenance of the genome and of chromosome forks [16], [17]. These three proteins accumulate at chromosomal forks in an SSB_Cter_-dependent manner, a discrete localization that does not depend on accidental fork arrest [8]. In addition, we have characterized a *B. subtilis* PriA mutant unable to interact with SSB, which was no longer targeted to active chromosomal forks and did not support replication restart unless overproduced. This underlines the direct benefit of pre-recruitment and targeting of PriA by SSB on active chromosomal forks: in anticipation of a requirement of PriA repair action, which can occur at any stage of genome replication [8]. Thus, an hypothesis raised by such a preparatory mode of PriA action at replication forks would also apply to the other SSB protein partners.

In this study, we have further defined the *B. subtilis* SSB_Cter_ interactome at active chromosomal forks. First, using cytological and biochemical approaches we have extended the number of *B. subtilis* SSB partner proteins targeted at forks to twelve, including RarA, SbcC and XseA, which are also present in *E. coli* but not previously known to interact with *_Ec_*SSB. Among the other proteins identified were key effectors of the RecFOR loading machinery for RecA. In addition, 3 others, including the DNA polymerase DnaE, appear to be specific to the *B. subtilis* SSB interactome. Paradoxically, although DnaE is one of the two essential *B. subtilis* DNA polymerases, this interaction is not essential since we have been able to delete SSB_Cter_ while retaining cell viability [8]. In parallel to this screening, we have undertaken detailed analysis of the multiple defects caused by the deletion of the SSB_Cter_ *in vivo*. Based on these results we propose an integrated model for replication fork rescue in which SSB coordinates the multiple processes potentially involved in a cascade-like manner. In an initial response to replication fork blockage, the system would first attempt to repair the damage and restart the stalled fork by the coordinated action of proteins present at the fork prior to its blockage. Failure to circumvent the blockage in this way would lead to a second set of responses, in particular the *de novo* loading of RecA on ssDNA at arrested forks by specific SSB-associated proteins. This would facilitate fork remodeling by homologous recombination (reviewed in [2]). Failure at this step would then lead to a more robust response by induction of the SOS system to provide increased levels of repair proteins to repair damaged forks.

## Results

### Defining the *B. subtilis* SSB_Cter_ interactome at chromosome replication forks

A remarkable feature of the *B. subtilis* SSB protein is that deletion of its C-terminal end is not lethal to the cell, in sharp contrast to that of *E. coli* [9]. This enabled the demonstration that PriA, RecG and RecQ proteins are targeted to active chromosome replication forks in *B. subtilis* in a manner which depends on the C-terminal region of SSB [8]. To identify additional proteins targeted to active chromosomal forks in the same way, we have extended these studies by using two *B. subtilis ssb* alleles truncated for the last 35 (*ssbΔ35*) or 6 (*ssbΔ6*) codons.

Candidate proteins were chosen according different criteria. Some were *E. coli* homologues already shown or proposed to interact physically with the *_Ec_*SSB_Cter_ (reviewed in [6]). Others were selected because of their sequence or functional homology with known partners of *B. subtilis* SSB. A third group included those already known to be present at active chromosomal forks. The final group comprised proteins selectively purified with *B. subtilis* SSB using the Tap-tag procedure [18] and identified by mass spectrometry.

All candidates were screened for localization at active replication forks as GFP fusions, expressed ectopically from the *amyE* locus, in SSB_Cter_ deletion strains and in the isogenic wild type strain (*ssb3^+^*). Most candidates were also screened for physical interaction with SSB as purified proteins *in vitro* or by Tap-tag analysis with the use of the SPA motif fused to their C-terminal end at their original genetic locus. The combination of these three approaches identified 9 additional proteins which together represent an extended view of the SSB_Cter_ interactome targeted to active *B. subtilis* chromosomal forks. The results of this screening are compiled in Table 1 and described in the following sections.

**Table 1:** The *B. subtilis* SSB interactome targeted to active chromosomal replication forks.

Protein partners of *B.subtilis* SSB	GFP foci formation^a^	*in vitro* interaction with SSB b	Tap-tag experiments	
			protein candidates^c^	SSB^d^
PriA*	+	+	-#	-#
RecQ*	+	+	+	+
RecG*	+	+	+	+
YpbB§/RecS§	+	+	ND/+	−/+
DnaE	+	+	-	-
SbcC§	+	ND	ND	-
RarA§	+	+	-	+
RecJ§ *	+	ND	+	+
RecO*	+	+	-#	-#
YrrC§	+	ND	ND	+
XseA§	+	ND	ND	+
Ung*	ND	ND	ND	+

In the first column are listed all *B. subtilis* candidate proteins found to belong to the SSB interactome targeted to active forks. The

*denotes proteins that were previously known to interact with SSB in *B. subtilis* or in other bacteria [6], [8].

a: + signifies that discrete GFP foci for each fusion protein observed in *ssb3+* cells were no longer detected in *ssbΔ35* cells.

b: + signifies that in pull-down and/or in gel-filtration assays most of the retention of candidate proteins by SSB is no longer seen with SSBΔ6.

c: + and - signify ability/inability of candidate proteins to capture SSB in Tap-tag experiments.

d: + and - signify detection/non detection of the protein candidate in the SSB-SPA purification experiment presented in Figure 3. −/+ indicates that RecS but not YpbB was detected. ND: not determined.

The

§ denotes GFP fusions that have not been tested functionally.

The

# denotes proteins naturally expressed at very low levels in the cell; this provides a simple explanation for inability to detect them in the Tap-tag experiment of SSB, and reciprocally, the non detection of SSB in the Tap-tag experiments performed with these proteins.

### RecS, a paralogue of RecQ in *B. subtilis*, interacts with SSB in association with YpbB

*B. subtilis* and closely related bacteria encode two RecQ homologues [19]. The first, initially annotated as YocI, was renamed RecQ since it is highly homologous to the single RecQ protein generally encoded in bacterial genomes (including *E. coli*). *B. subtilis* RecQ co-localizes with active replication forks in an SSB_Cter_-dependent manner and Tap-tag analysis of RecQ provided further evidence for its ability to interact unaided with SSB [8]. The second RecQ homologue, RecS (also annotated as YpbC), is smaller. The RecQ family is typified by 8 helicase motifs. In both RecQ and RecS, they are located towards the N-terminal (Nter) region and are followed by a more divergent C-terminal (Cter) region. In *_Ec_*RecQ, the latter region carries the site of interaction with SSB [20], [21].

**Figure 1:**
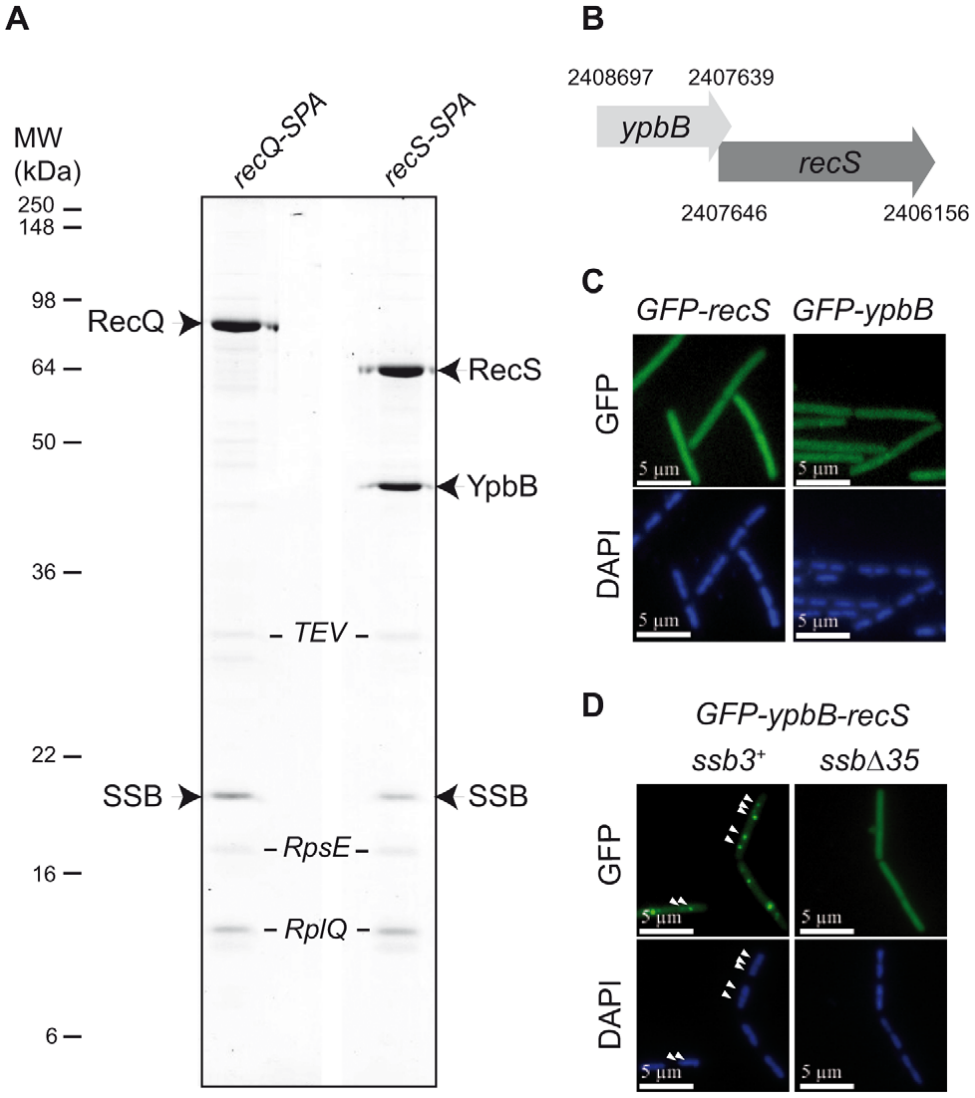
RecS assembles with YpbB into a complex targeted to active chromosomal replication forks *via* the SSB_Cter_. (A) Tap-tag purification of RecS-SPA and RecQ-SPA fusions. Purified proteins have been separated on a 12.5% SDS-PAGE, revealed by Coomassie staining and identified by MALDI-TOF analysis following in-gel trypsinolysis. The ribosomal RpsE and RplQ proteins are prominent contaminants systematically recovered by this purification method with *B. subtilis*. These contaminants have been indicated in italic, as a matter of distinction with the others considered as specific partners of RecQ or RecS proteins. All bands that have given a positive spectrum in the predicted *B. subtilis* protein database (excepted for the TEV protease added during the purification) have been accordingly annotated on the picture of the gel. (B) Genetic organization of the *ypbB-recS* region. The Comprehensive Microbial Resource [54] was used to identify coordinates of the genes contained in the Genbank *B. subtilis* genome sequence version AL009126. (C) and (D) GFP (green), and DAPI (blue) fluorescent signals from cells expressing the GFP protein fusions indicated above each panel. In (D), white arrowheads point to GFP foci on the DAPI-stained nucleoid of 2 representative *ssb3^+^* cells (see Table S1 for statistical analysis of this foci distribution).

As shown in Figure 1A, Tap-tag analysis of RecS revealed a prominent interaction with SSB and a protein of unknown function, YpbB, encoded immediately upstream of *recS*. The stop codon of *ypbB* overlaps the start codon of *recS*, suggesting translational coupling of the two proteins (Figure 1B). In the few bacterial species encoding a *ypbB* homologue, this invariably appears upstream of a *recS* homologue in a common operon (see Figure S1). In the Tap-tag experiments, RecS and YpbB appear in almost equimolar amounts after purification, as does the co-captured SSB (Figure 1A). In addition, the cellular concentrations of RecS and RecQ appear similar (between 150 and 300 copies per cell) as judged by western-blotting of total protein extracts of cells expressing the RecS-SPA or RecQ-SPA fusions and probed with anti-Flag antibodies against the SPA motif (not shown). To test whether RecS and/or YpbB are targeted to chromosomal replication forks, we first constructed N-ter GFP fusions of each gene individually at *amyE*. Both GFP-RecS and GFP-YpbB fusion proteins appeared largely dispersed throughout the cell (Figure 1C) although some cells were found to exhibit tiny foci on their nucleoid (Table S1). In view of their tandem genetic configuration, it seemed possible that both might be required for correct targeting. We therefore inserted a construction (*GFP-ypbB/recS*) including both genes at the *amyE* locus, to retain potential translational coupling. As shown in Figure 1D, all *ssb3^+^* cells carrying this construct exhibit a regular GFP focus pattern identical to that observed previously with *GFP-recQ* ([8]; Table S1). The simplest explanation for this, as implied by the Tap-tag analysis (Figure 1A), is that YpbB and RecS assemble into a single complex able to interact with SSB, resulting in its targeting to active chromosome replication forks. Biochemical evidence for physical interaction between YpbB and RecS comes from our attempts to purify them from *E. coli* (described in Text S1): YpbB could not be prepared as a soluble protein alone but only as a stable complex with RecS. Furthermore, the YpbB/RecS complex, but not RecS alone, interacts physically with SSB (Figure S2). Finally, no localization of GFP-YpbB was observed in *ssbΔ35* and *ssbΔ6* cells (Figure 1D and Table S1).

Altogether, these results show that YpbB is targeted to active chromosomal forks in an SSB_Cter_-dependent manner. They also indicate that the GFP-YpbB foci depend on RecS, with which YpbB forms a stable complex. Reciprocally, RecS could also be present at forks via its association with YpbB. The detection of RecS in the Tap-tag of SSB argues for this is the case (see below).

### PcrA and DinG DNA helicases do not belong to the interactome of SSB

To test whether localization at active forks is a property shared by other DNA helicases known to act in repair of arrested replication forks, we analyzed PcrA [22]. A functional GFP-PcrA fusion did not form foci in growing cells but appeared to localize non-specifically to the nucleoid (Figure S3A). In addition, SSB was not detected among the proteins that co-purified with PcrA in Tap-tag experiments (Figure S3B). The Tap-tag is nevertheless validated by the recovery of 2 known partners of PcrA with the functional PcrA-SPA fusion: RNA polymerase [23], and RecA [24], [25]. Furthermore, we did not detect an interaction between purified SSB and PcrA (active as a DNA helicase) in a specific SSB pull-down assay (Figure S4C) detailed below. A lack of discrete targeting in the cell was also observed for the widespread DinG DNA helicase (Figure S3A), recently demonstrated in *E. coli* to function in concert with Rep or UvrD (the functional equivalents of PcrA; [26], [27]) in resolving accidents caused by collision between the replication and transcription machineries. In addition, SSB was not detected in the Tap tag of DinG (Figure S3B). Most of the proteins co-purified with DinG were ribosomal proteins, indicative for a putative role of DinG in translation and/or ribosome biogenesis in *B. subtilis*.

Thus, anchorage to active chromosomal forks visualizable by focus formation would not be a hallmark of all effectors of DNA replication rescue. We could not exclude, however, that specific interactions of PcrA and DinG with one component of the fork might occur without being strong or cumulative enough to generate a detectable focus.

### The essential DNA polymerase DnaE accumulates at active chromosomal DNA replication forks in an SSB_Cter_–dependent manner

**Figure 2:**
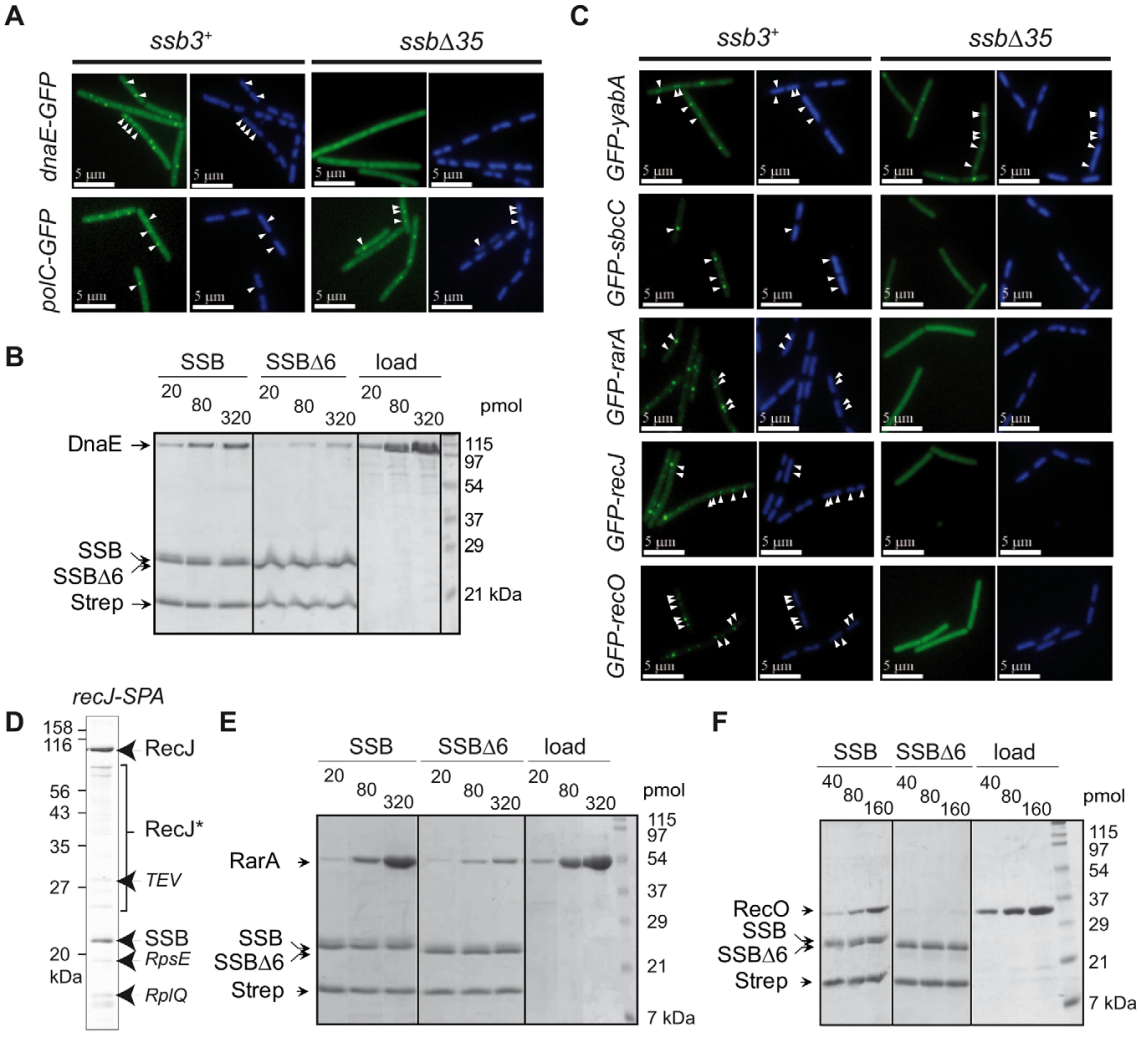
Extending the composition of the SSB_Cter_–dependent interactome. (A), (C) GFP (green), and DAPI (blue) fluorescent signals from cells expressing GFP fusions (listed on the left of each set of panels) in *ssb3^+^* or *ssbΔ35* cells. White arrowheads point to GFP foci visible on the DAPI-stained nucleoid of two representative cells (see Table S1 for statistical analysis of this foci distribution). (B), (E), (F) *In vitro* binding assay between purified DnaE, RarA or RecO respectively (at the amounts indicated on the top of the gels) and 80 pmol of tetramer of SSB or SSBΔ6 molecules (see Materials and Methods for details). Proteins are indicated on the left of the 14% SDS-PAGE gel stained by Coomassie-blue. Strep stands for streptavidin. The ‘load’ on the right side of each gel corresponds to the range of each protein tested for interaction on the SSB (SSBΔ6)-coated beads. (D) Tap-tag of a RecJ-SPA fusion protein (the experiment was done and presented as in Figure 1). RecJ* indicates degradation product of RecJ-SPA.

We previously showed that DnaX, a homologue of the *E. coli* Holopolymerase III (*_Ec_*PolIII) τ subunit, still formed foci in *ssbΔ35* dividing cells [8]. This is also true for other components of *_Ec_*PolIII conserved in *B. subtilis*, i.e. HolA, HolB and DnaN, as well as for the replicative DNA helicase, DnaC (Table S1). We could not test the primase, DnaG, since neither N- nor C-terminal GFP fusions gave rise to discrete foci in wild-type *B. subtilis* cells [10]. *B. subtilis* encodes two DNA polymerases essential for genome duplication, PolC and DnaE [28]. Both are homologous to the single essential *E. coli* DNA polymerase, *_Ec_*DnaE. GFP fusions to PolC and DnaE were both shown to localize at active chromosomal forks [28], [29]. Remarkably, and in sharp contrast with PolC-GFP, we found that DnaE-GFP did not form foci in *ssbΔ35* cells (Figure 2A and Table S1) nor in *ssbΔ6* cells (Table S1). This suggests that DnaE accumulates at active chromosomal forks *via* a physical interaction with SSB whereas PolC does not. Tap-tag analysis of DnaE was not informative, since only the DnaE-SPA prey was recovered (not shown). We therefore explored the potential interaction between DnaE and SSB *in vitro* with purified recombinant proteins. We used a pull-down assay based on magnetic beads coated with ssDNA fully bound by purified SSB or SSBΔ6 (or SSBΔ35, which behaved as SSBΔ6; Figure S5) to test specific interaction between a protein and the SSB_Cter_. This assay was validated with RecQ and RecG (see Figure S4A and S4B; Figure S5). Purified DnaE was found to interact with the SSB_Cter_ in this assay and poorly with SSBΔ6 and SSBΔ35 (Figure 2B and Figure S5), supporting the notion that the interaction between DnaE and SSB is direct and accounts for the accumulation of DnaE-GFP at active chromosomal forks.

### Extending the *B. subtilis* SSB interactome at active forks

We next examined the localization of other proteins known or expected to co-localize with *B. subtilis* chromosomal forks but not essential for their propagation. These included SbcC, a subunit of the heterodimeric SbcCD nuclease that acts specifically on ssDNA palindromic structures [30]; YabA, a negative regulator of initiation of DNA replication at *ori*C in *B. subtilis* [31]; RarA, which, in *E. coli,* is required for RecA loading on arrested chromosomal forks [32]; and RecO and RecJ, which are also involved with RecA loading at arrested forks in concert with RecF, RecR and RecQ.

Among these, only YabA was found to localize in *ssbΔ35* cells (Figure 2C, Table S1). Since YabA localizes at forks in a DnaA- and DnaN-dependent manner [33], this result implies that DnaN and DnaA act in *ssbΔ35* cells as in wild-type cells.

GFP-SbcC was previously shown to co-localize with *B. subtilis* replication forks [10]. Here we find that this localization is dependent on the C-terminal domain of SSB (Figure 2C and Table S1).

The three others candidate proteins, i.e. RarA, RecO, and RecJ, which did not localize in *ssbΔ35* cells in contrast to wild type *ssb3* ^+^ cells (Figure 2C and Table S1), were found to interact physically with SSB. This was demonstrated, by Tap-tag analysis in the case of RecJ (Figure 2D), by pull-down and gel filtration assays for RarA (Figure 2E and Figure S6) and by pull-down assays for RecO (Figure 2F). The precise targeting of the functional GFP-RecO fusion to active forks (and not to ssDNA gaps that could be formed elsewhere on the genome) was confirmed by its co-localization with the replisome protein DnaX (Figure S7).

These results imply that although RecA is not normally present at active forks [34], these are equipped with key components of the RecFOR machinery (i.e. RecO, RecJ, RecQ and RarA) permitting recruitment of RecA at replication forks upon accidental arrest [34].

### Tap-tag analysis of *B. subtilis* SSB

**Figure 3:**
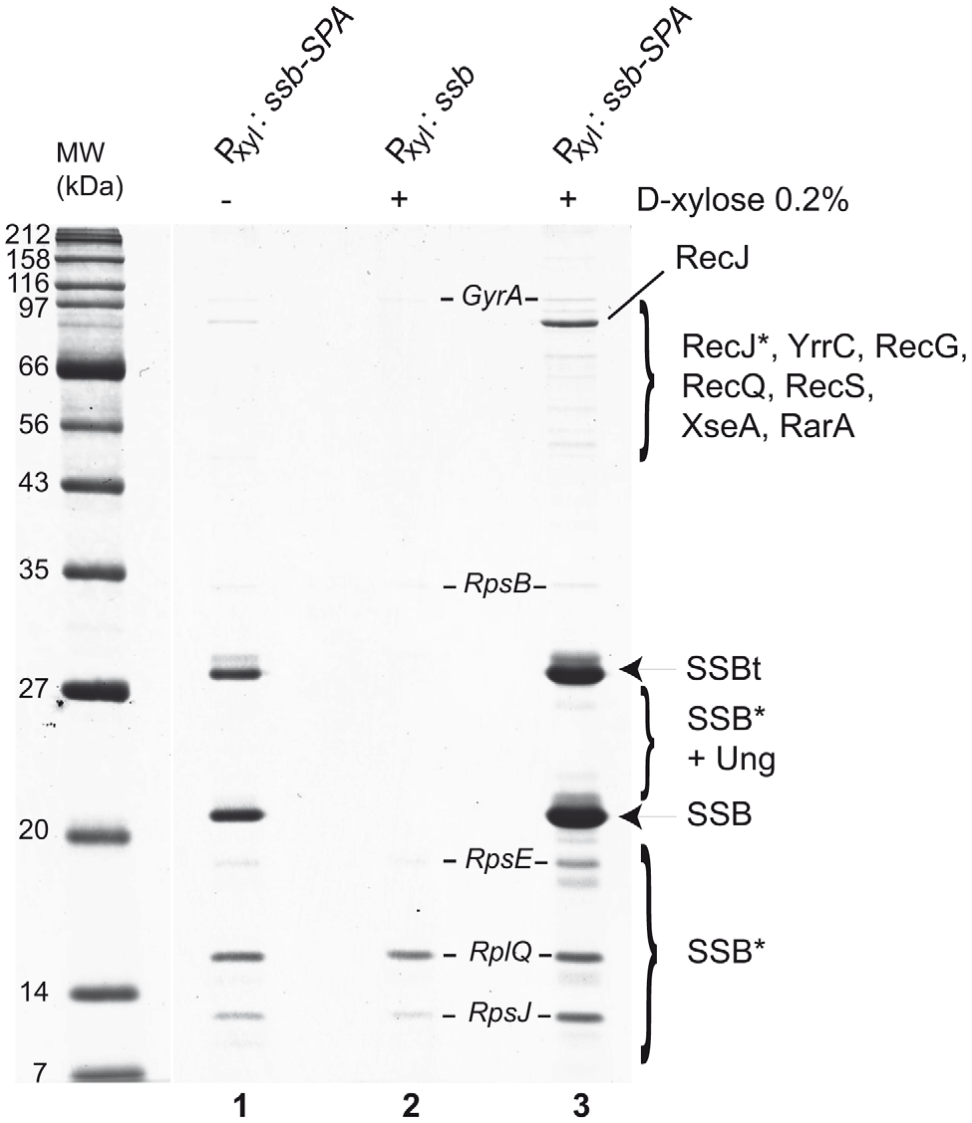
Tap-tag of *B. subtilis* SSB. Cells harbouring the P_xyl_: *ssb-SPA* construct at *amyE* were grown to mid-log phase in LB medium without (−) or with (+) 0.2% D-xylose (lanes 1 and 3). The SSB-SPA fusion was purified and analyzed as described in Figure 1. The isogenic strain containing the wild type *ssb* instead of the *ssb-SPA* construct at *amyE* was treated in the same way (lane 2). All proteins identified by MALDI-TOF analysis of Coomassie-stained bands have been reported on the gel picture. RecJ* indicates RecJ degradation products. SSBt stands for the SSB-SPA purified after cleavage by the TEV protease. SSB* indicates degradation products of SSBt or of wild-type SSB.

To identify a more complete repertoire of SSB partners, we used SSB as a prey in Tap-tag analysis. However, in contrast to *_Ec_*SSB [35], we were unable to construct an SSB-SPA fusion at the *ssb* locus suggesting that capping SSB_Cter_ with the SPA motif inactivates SSB and leads to cell lethality. We therefore inserted the *ssb-SPA* construct under the P_xyl_ promoter at *amyE* to generate mixed SSB complexes composed of both SSB-SPA and wild-type SSB subunits. Their selective capture, via the SSB-SPA component should permit the co-capture of protein partners interacting with the uncapped wild-type SSB subunits. Ectopic expression of SSB-SPA had no observable negative effect on cell growth (not shown). As shown in Figure 3, wild type SSB and SSB-SPA subunits were recovered in equal amounts from cells grown with (lane 3) or without (lane 1) D-xylose induction of SSB-SPA expression. As expected, the total yield of the hetero-tetrameric SSB/SSB-SPA complexes was higher with than without D-xylose.

Many proteins were observed to co-purify with SSB/SSB-SPA complexes (Figure 3). These included those reproducibly recovered in other Tap-tag experiments performed with other *B. subtilis* proteins (e.g. GyrA and many ribosomal proteins) and were not considered further. The others appeared to be specific partners of SSB. They were not observed in control experiments where the ectopic *ssb-SPA* was replaced with a wild type *ssb* allele (lane 2). In addition, their levels were increased when SSB-SPA expression was induced (compare lanes 1 and 3). Many of these proteins (e.g. RecJ, RecQ, RecG, and RarA) were identified in the experiments described above as direct partners of *B. subtilis* SSB. Very few peptides of RecS were unambiguously detected by mass spectrometry. As reported above, RecS alone could not stably interact with SSB *in vitro* but could do so in a complex with YpbB (Figure S2). YpbB was not detected in the Tap tag of SSB, possibly because the number of YpbB molecules recovered was below the level of detection by mass spectrometry, RecS being very close to this limit. The Ung protein was identified in the Tap tag of SSB. Ung was identified previously as a partner of *_Ec_*SSB [36]. However, some known SSB partners, such as RecO, PriA, DnaE, SbcC and YpbB, were absent. This is probably due to differences in affinity between SSB and each of its partners and to variation in their natural cellular levels since some are known to be present in very low amounts (for instance, ∼50 copies of PriA per cell; [15]). The same dual explanation may also account for the differential yield of some SSB partners recovered in the experiment e.g. RecJ which is by far the most abundant protein co-purified with the SSB/SSB-SPA complex (Figure 3).

We also identified several new candidate partners. These included XseA, the large subunit of ExoVII, and YrrC, a protein of unknown function conserved in gram positive bacteria and predicted to be a helicase/nuclease by sequence analysis. GFP fusions to XseA and YrrC were also both found to form foci on the nucleoid of *ssb3^+^* cells but not in *ssbΔ35* cells (Table S1).

This screening identified a repertoire of 12 proteins belonging to the *B. subtilis* SSB interactome escorting active chromosomal forks. Together, these proteins fulfill a large variety of functions concerned with DNA processing. As a result, the SSB_Cter_ emerges as a central hub of DNA processing functions at chromosomal replication forks, where SSB naturally concentrates in the cell.

### Growth and cellular defects of *B. subtilis ssbΔ35* mutant cells

**Figure 4:**
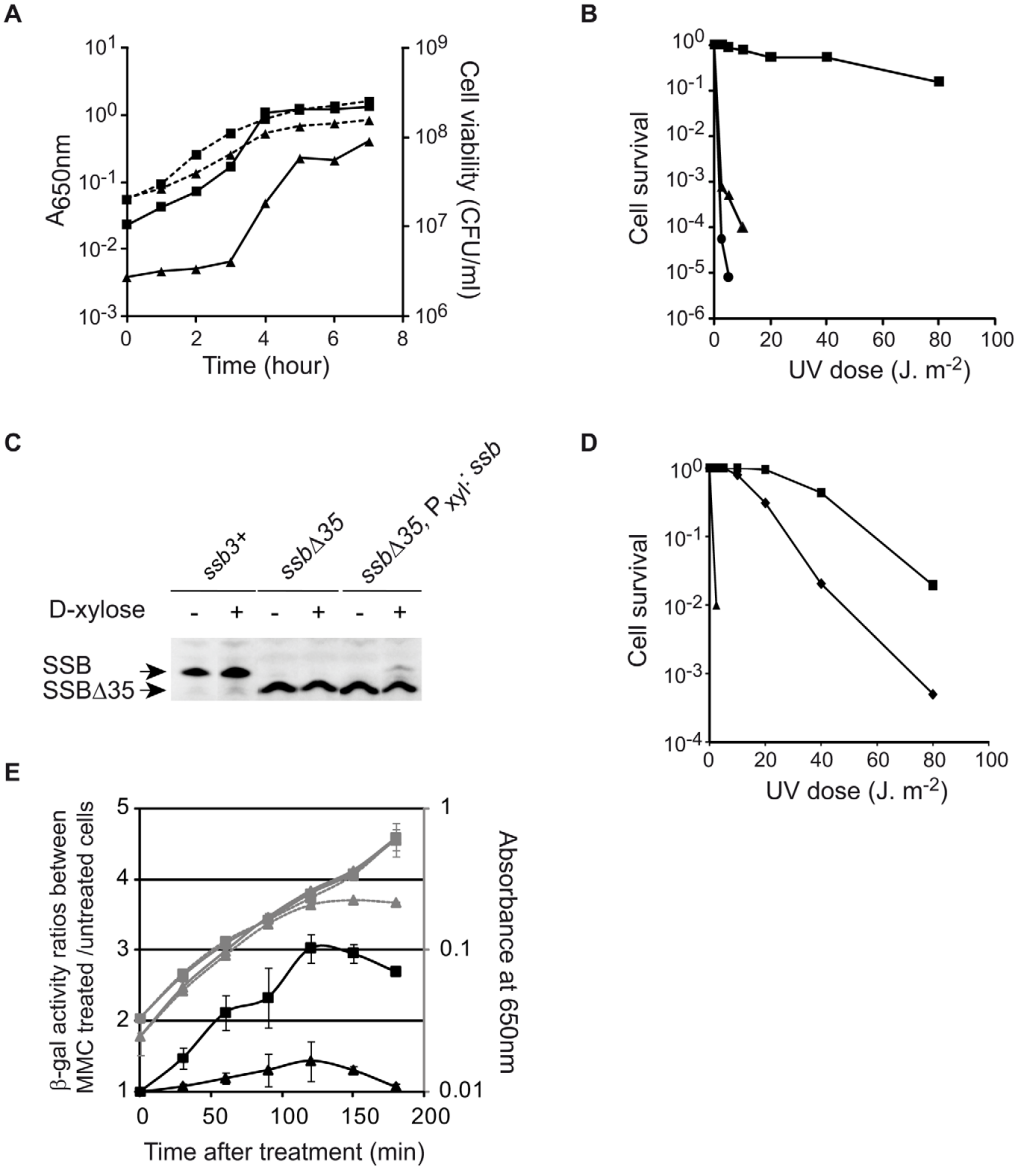
The SSB_Cter_ is crucial for optimal cell growth and genome maintenance. (A) Growth kinetics of *ssbΔ35* (triangles) and *ssb3^+^* (squares) strains in LB supplemented with erythromycin and IPTG at 37°C. Growth was followed by monitoring A_650nm_ (dashed lines) and Colony Forming Unit/ml (CFU, solid lines) as a function of time. (B) UV sensitivity of *ssb3^+^* (squares), *ssbΔ35* (triangles) and *recA* ^−^ (circles) cells grown in LB at 37°C with appropriate antibiotics. (C) Western blot analysis of SSB and SSBΔ35 proteins in *ssb3^+^, ssbΔ35* and *ssbΔ35* P_xyl_:*ssb* cells grown in presence (+) or not (−) of D-xylose in the medium. Analyses were done as described in Materials and Methods. Signals corresponding to SSB and/or SSBΔ35 are indicated on the left of the membrane. (D) UV sensitivity of *ssb3^+^* (squares), *ssbΔ35* (triangles) and *ssbΔ35* P_xyl_ *:ssb* (diamonds) cells grown in LB supplemented with erythromycin, IPTG and 0.2% D-Xylose. For A, B and D, one typical experiment is reported (they have been reproduced at least 3 times with independent clones and led to similar results). (E) Measurement of the MMC-induced SOS response in *ssb3^+^*/P_lexA_:*lacZ* (squares) or *ssbΔ35/*P_lexA_:*lacZ* (triangles) strains. Absorbance at 650 nm of treated (gray dashed lines) and untreated (gray continuous lines) cultures was monitored after MMC treatment (40 ng/ml added to the cultures at time 0 min). β-galactosidase specific activities were measured using extracts prepared from treated and untreated cells at different times after addition of MMC (see Materials and Methods). Ratios of β-galactosidase activities of treated to untreated cells for each time point are plotted (black lines). Error bars indicate the standard deviation from the mean calculated from two independent experiments.

**Figure 5:**
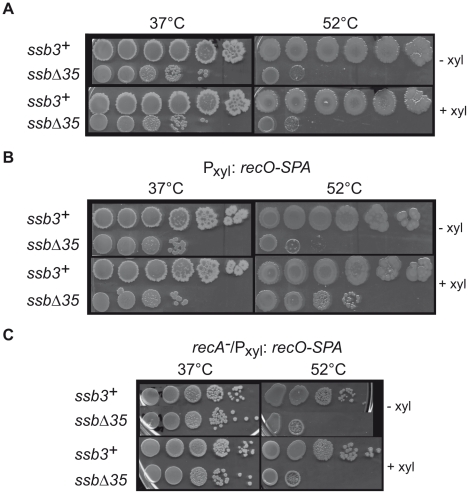
RecO overexpression suppresses temperature-sensitive growth of *ssbΔ35* cells. (A) *ssb3^+^* and *ssbΔ35* cells (as indicated on the left of the pictures) were grown to mid-log phase at 37°C in LB with (+ xyl) or without (− xyl) 0.2% D-xylose, serially 10 fold diluted, spotted on agar plates containing the same medium and incubated at 37°C or 52°C. (B) Identical spot assay as in (A), with the P_xyl_:*recO-SPA* construct inserted at *amyE*. (C) Identical spot assay as in (B) in a *recA^−^* background.

While deletion of the SSB_Cter_ is not lethal, *ssbΔ35* cells show viability defects. They exhibit a ∼5-10 fold lower plating efficiency during growth in rich medium, i.e. under fast growing conditions (Figure 4A), as well as in minimal medium (not shown), and smaller colonies on solid medium (Figure 5A). The reduced viability was also directly inferred by observation of exponentially growing cells (in rich medium) under the microscope where up to 15% of *ssbΔ35* cells show various kinds of cellular and/or nucleoid morphological defects (i.e. distribution, shape, length, segregation; see Figure S8). Similar observations were made with the *ssbΔ6* strain (Figure S9A and S9B and not shown).

### The SSB_Cter_ is crucial for rescuing the damaged genome

The SSB_Cter_ interactome includes many proteins involved in maintaining genome integrity. The importance of the SSB_Cter_ might therefore be expected to be more pronounced under growth conditions that are stressful for the genome. Indeed, *ssbΔ35* and *ssbΔ6* cells are nearly as sensitive to UV irradiation as *recA^−^* cells (Figure 4B and Figure S9C). Similarly, both mutants are also more sensitive to Mitomycin C (MMC) than the *ssb3^+^* strain (Figure S9D).

To investigate further the intracellular role of the SSB_Cter_, we examined the effect of complementation of the defects of *ssbΔ35* cells by ectopic expression of wild-type SSB at *amyE* in a controlled manner from the P_xyl_ promoter. Upon induction with D-xylose production of SSB from the P_xyl_ promoter was ∼10% that of the natural SSB level (Figure 4C; compare lane 2 with lane 6). This low concentration was, however, sufficient to fully suppress the plating defect of the *ssbΔ35* strain (not shown). It also fully restored UV resistance at doses up to 10 J/m^2^ (Figure 4D). Above this dose, the cells exhibited sensitivity intermediate between that of *ssb3^+^* and *ssbΔ35* cells indicating that the intracellular concentration of SSB_Cter_ is determinant for an optimal response to DNA damage.

We next investigated whether SOS, a well known cellular response to DNA damaging agents, could be triggered in *ssbΔ35* cells. In *B. subtilis*, the SOS system is regulated by RecA-induced auto-cleavage of the LexA repressor. We used a P_lexA_:*lacZ* construct as a reporter of SOS activity and another DNA damaging agent, MMC, as an inducer [37]. The MMC-induced SOS response was dramatically reduced in *ssbΔ35* cells compared to *ssb3^+^* cells (Figure 4E). These results therefore underline a pivotal role for the SSB_Cter_ in triggering the SOS response.

### SSB_Cter_ deletion mutants are temperature-sensitive

Another notable defect of *ssbΔ35* strains is their temperature-sensitive growth, as measured by plating assay (Figure 5A). This lethality is fully corrected by SSB expression induced from the ectopic P_xyl_:*ssb* (not shown). Since some SSB partners are independently important for cell viability, we tested whether the temperature sensitivity of *ssbΔ35* cells could be corrected by increasing individual expression of these partners placed under the P_xyl_ promoter from the *amyE* locus. Overexpression of DnaE or PriA, the two most important components of the SSB interactome for cell viability, did not alleviate the temperature sensitivity of *ssbΔ35* cells (not shown). Unexpectedly, however, induced expression of RecO (as a functional RecO-SPA fusion) did so (Figure 5B). In contrast, the plating defect characteristic of the *ssbΔ35* strain observed at permissive temperature is not corrected by RecO overexpression (Figure 5B).

RecO is a recombination mediator protein that, with RecR and RecF, directs loading of RecA onto ssDNA coated by SSB [38]. We therefore tested whether suppression of *ssbΔ35* temperature sensitivity by overexpression of RecO was dependent on RecA. We introduced the *recA::tet* allele into the *ssbΔ35* and *ssb3^+^* strains carrying the P_xyl_:*recO-SPA* cassette. Inactivation of *recA* in the *ssb3^+^* strain provoked weak temperature sensitivity (Figure 5C). Disruption of *recA* prevented RecO suppression of *ssbΔ35* temperature sensitivity (Figure 5C). This implies that RecA loading on ssDNA is needed for this suppression. The formation of a RecA-ssDNA nucleofilament is the pre-synaptic intermediate of homologous DNA recombination and the inducing signal of SOS, which we have shown above to be defective in MMC-treated *ssbΔ35* cells (Figure 4E). However, individual overexpression of RecO neither restored SOS induction by MMC in this mutant, nor suppressed its sensitivity to MMC (not shown).

Thus, the suppression is not solely due to RecO action, but also relies on RecA, leading to the proposal that it proceeds through the RecO-dependent loading of RecA on ssDNA.

### The SSB_Cter_ is needed for supporting genomic DNA replication

**Figure 6:**
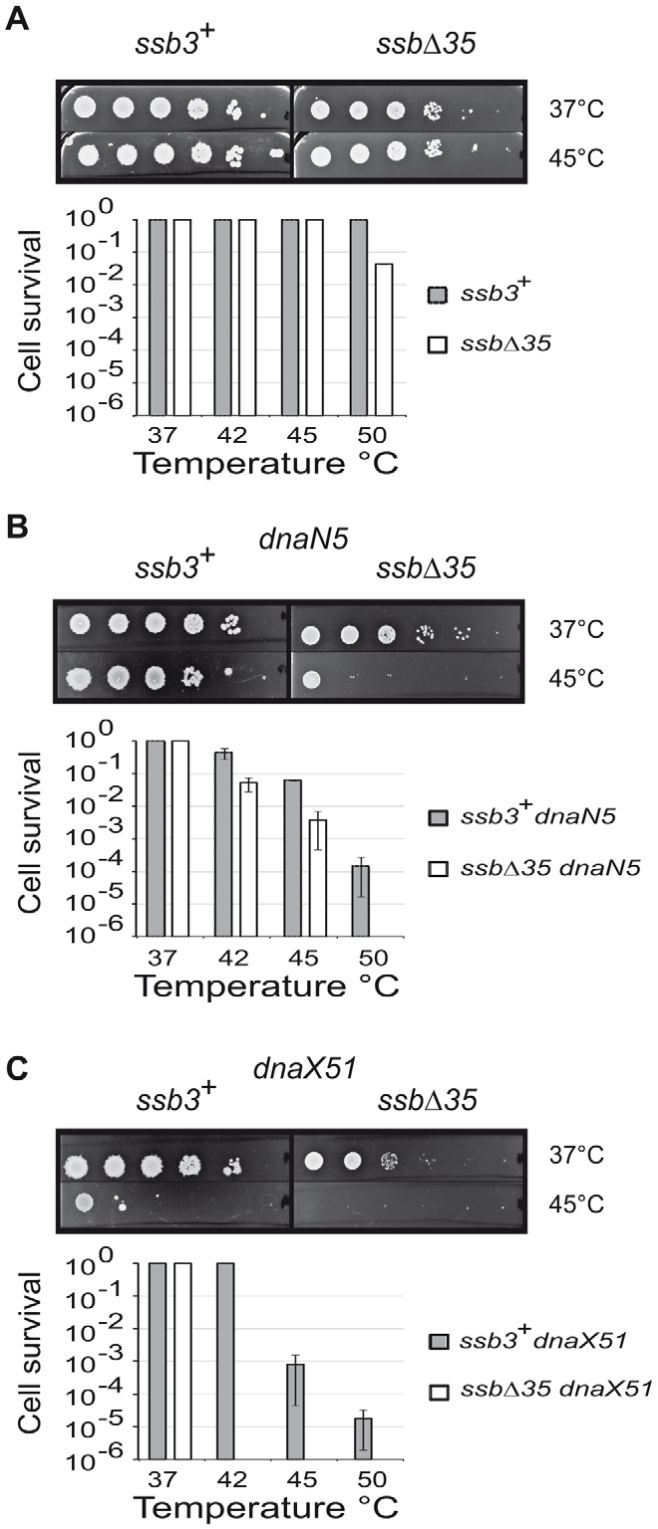
The SSB_Cter_ is crucial for the rescue of arrested DNA replication forks. Cells grown to mid-log phase at 30°C in LB (supplemented with IPTG and erythromycin) were serially 10 fold diluted and spotted on agar plates of the same medium, and incubated at the indicated temperatures. The relevant genotype of the strains analyzed is indicated on the top of the plates, corresponding to the growth at 37°C and 45°C. (A) *ssb3^+^* and *ssbΔ35* strains (B) *ssb3^+^ dnaN5* and *ssbΔ35 dnaN5* strains and (C) *ssb3^+^ dnaX51* and *ssbΔ35 dnaX51* strains. Cell survival was determined at different temperature of incubation as indicated in the diagrams. Error bars indicate the standard deviation from the mean calculated from three independent experiments.

The previous experiments demonstrated that SSB_Cter_ was required for repair of lesions throughout the genome. They did not address the question of whether the SSB_Cter_ specifically assists chromosomal fork progression. To specifically stress the replication fork, we used two *B. subtilis* strains bearing temperature sensitive alleles, *dnaN5* and *dnaX51*, whose products are exclusively associated with the replisome [39] and analyzed how deletion of the SSB_Cter_ affects their viability. The *ssbΔ35* allele, genetically linked to the erythromycin resistance marker (Ery^R^), was introduced by transformation at low temperature into strains carrying the replication mutations. An isogenic Ery^R^ *-*linked *ssb3^+^* allele was also used as a control. Viable Ery^R^ clones were obtained upon transformation at 30°C of the *dnaN5* and *dnaX51* strains with the *ssbΔ35* and *ssb3^+^* alleles. The *ssbΔ35 dnaN5* and *ssbΔ35 dnaX51* recombinants exhibited the characteristic plating and growth defects of the *ssbΔ35* strain (Figure 6). Interestingly, they were both significantly more temperature sensitive for growth than their *ssb3^+^* counterparts (Figure 6A–6C). Thus the SSB_Cter_ is crucial for growth of *ssb3^+^ dnaN5* and *ssb3^+^ dnaX51* cells at semi-permissive temperatures. In addition, we also found that *ssbΔ35* cells were markedly more sensitive than *ssb3^+^* cells to DNA replication stresses induced either by hydroxyurea, which diminishes the dNTP pools, or by HPUra, an antibiotic that specifically inactivates the essential DNA polymerase, PolC (not shown).

The exact nature of the defects provoked by a dysfunction of the replisomes made with the mutated DnaN or DnaX proteins is not known. These defects could be either fork arrest or lesions left by continuing forks. These results provide evidence that the SSB_Cter_ is crucial for ensuring the proper duplication of a genome damaged by stresses that specifically impair the replisome.

## Discussion

In this study, we have extended the number of known *B. subtilis* proteins involved in the SSB_Cter_ interactome and targeted to active chromosomal DNA replication forks. It includes at least 9 additional members (Table 1), in addition to the PriA, RecG and RecQ DNA helicases [8]. Collectively these constitute a multipurpose DNA processing toolbox able to unwind, replicate or cleave DNA and to promote homologous DNA recombination. Replication forks can consequently marshal a large repertoire of enzymes for their rescue or for preventing their accidental arrest. This is revealed by an increased sensitivity to a variety of replication stresses of *B. subtilis* cells carrying an *ssb* allele truncated for its C-terminal domain, SSB_Cter_ (Figure 6). Thus, in addition to assisting the activities of the enzymes of the replisome by polymerizing along ssDNA *via* its Nter domain, SSB provides constant support for fork progression *via* its Cter domain by mediating multiple DNA transactions. It does so by concentrating a specific subset of proteins of the DNA recombination, repair and replication machineries at active forks.

### The SSB_Cter_ acts as a general hub of DNA maintenance proteins

SSB is not the only source of accessory proteins at the replication fork. DnaN and the replicative helicase also act as anchors for distinct replication accessory proteins (for reviews, see [5], [40]). While DnaN and the replicative helicase are expected to be confined to replication forks, the spectrum of SSB activity on the genome could be larger since its localisation is primarily determined by availability of ssDNA. Assembly of the SSB interactome at a precise site on the genome is nevertheless expected to be qualitatively and quantitatively modulated by the length of the ssDNA available for SSB polymerisation. Indeed, the local concentration of SSB_Cter_ will increase with the length of the SSB-ssDNA nucleofilament. With an average length of 1 kb for single strand DNA on the lagging strand template at an active bacterial DNA replication fork and a binding mode of SSB of ∼65 nts per tetramer, a minimum of ∼60 copies of SSB_Cter_ may be present at each fork. This would generate a filament capable of attracting many molecules of the different SSB interactome members. Consequently, chromosomal DNA replication forks constitute permanent subcellular sites for assembling the SSB interactome in dividing cells. In addition, all DNA processes that generate stretches of ssDNA accessible to SSB tetramers are expected to produce such centers for the SSB_Cter_ interactome anywhere on the genome (as in the case of the repair of DNA double-strand breaks). Thus, the DNA toolbox associated with the SSB_Cter_ should not be considered as exclusively devoted to the progression of replication forks. In contrast to DnaN and the replicative helicase, the SSB_Cter_ may therefore be a general determinant for the maintenance of genome integrity.

### The SSB_Cter_ interactome is specific

Comparison of the SSB_Cter_ interactome of *E. coli* (compiled in [6]) and that determined here for *B. subtilis* provides several important general conclusions concerning SSB_Cter_ function.

Homologues of prominent *_Ec_*SSB partners such as PriA, RecQ, RecG, RecJ, RecO and Ung which are conserved in *B. subtilis* (and generally widespread in bacteria) have also been demonstrated to interact with *B. subtilis* SSB. This points to a strong selective pressure in maintaining such a conserved and abundant SSB_Cter_ interactome. In this study, we have identified additional *B. subtilis* SSB partners (i.e. RarA, SbcC and XseA) also widely conserved in bacteria (including *E. coli*) but not yet identified as part of the *_Ec_*SSB interactome. Conversely, we have not yet identified other known conserved *_Ec_*SSB partners, such as DnaG primase and Topoisomerase III [41], [42], in the SSB interactome of *B. subtilis*. Further experiments will be required to determine whether these proteins are indeed members of the SSB interactome.

*B. subtilis* SSB partners that are not widely conserved in bacteria have also been identified, e.g. YrrC (a putative helicase/nuclease encoded in the genomes of gram positive bacteria also annotated as RecD) and the YpbB/RecS complex (see Figure S1). Conversely, *_Ec_*SSB partners, such as the χ subunit of the *_Ec_*Holopolymerase III [43], [44], have no equivalent in *B. subtilis*. Thus, the interactome of SSB also includes some specific proteins representative of subgroups of bacteria. This reveals particular needs in genome metabolism, suggesting that not all bacteria require these functions and/or have evolved distinct alternative strategies to execute identical functions.

Another specific part of the *B. subtilis* SSB_Cter_ interactome is the replisomal DNA polymerase DnaE [28]. Neither its homologue PolC, nor any other known proteins of the *B. subtilis* replisome, depend on the SSB_Cter_ for their targeting to active chromosomal forks (Table S1). DnaE remains essential for viability of *ssbΔ35* cells (not shown). Interestingly, it has recently been shown that the essential role of DnaE in the replisome is to elongate a short DNA stretch on the RNA primers synthesized by the DnaG primase before a hand-off to the bona fide replicative polymerase, PolC [45]. Thus, DnaE has presumably evolved distinct interactions with the replisome to functionally link the activities of DnaG and PolC. However, these interactions are not strong enough to produce detectable fluorescent foci with the DnaE-GFP fusion at active forks in *ssbΔ35* cells. In line with this reasoning, the DnaE interaction with the SSB_Cter_ might serve an additional role. *E. coli* encodes a single DNA polymerase of the DnaE family, which is not part of the *_Ec_*SSB interactome [6]. In contrast, the *_Ec_*DNA polymerase II (*_Ec_*PolII) has been found to interact with the *_Ec_*SSB [46]. *_Ec_*PolII is not essential for the cell, is involved in distinct pathways of replication re-activation and belongs to the *E. coli* SOS system [47]. Remarkably, *B. subtilis dnaE* also belongs to the SOS regulon [48]. This raises the possibility that *B. subtilis* DnaE might also be involved in certain fork maintenance pathways, as demonstrated for many members of the SSB interactome.

### The SSB_Cter_ is needed for RecA loading

A central piece of the SSB interactome is RecO, which acts with RecR and RecF to direct the loading of RecA on SSB-coated ssDNA [49]. Temperature sensitivity of *ssbΔ35* cells can be suppressed by RecO overexpression, and in a RecA-dependent manner. This shows that ssDNA accessible to RecA is generated in *ssbΔ35* cells at high temperature, and that RecA then mediates cell rescue. It also reveals a RecO dysfunction in the *ssbΔ35* strain, which can be compensated by increasing its cellular concentration. This parallels the results obtained previously with a PriA mutant unable to interact with SSB, whose inefficiency in directing replication restart was compensated by its overexpression [8]. Thus, a consequence of deleting SSB_Cter_ is a reduction in the activities of certain of its partners, resulting from loss of SSB_Cter_-assisted and targeted recruitment to their sites of action on the genome.

Importantly, RecO overexpression does not suppress the growth defect of *ssbΔ35* cells observed at permissive temperature. This points to the importance of the other SSB partners for sustaining optimal growth of wild-type cells. The growth defect of *ssbΔ35* cells could not be corrected by the overexpression of DnaE or of PriA alone; these are the two proteins of the SSB interactome known to be essential for growth (in rich medium in the case of PriA; [15]). Thus, it is possible that more than one SSB partner must be overexpressed to circumvent this defect, if this stems solely from the loss of their SSB-assisted targeting in the cell. Clearly, more work is needed to understand the growth defect caused by the deletion of the SSB_Cter_.

Another marked defect of *ssbΔ35* cells at permissive temperature is their inefficiency in inducing the SOS response upon treatment by MMC. This reflects a failure to generate the RecA-ssDNA filament which would normally act as a triggering signal. The RecFOR apparatus is needed for the MMC-mediated SOS induction in *B. subtilis* [50]. However, RecO overexpression in MMC-treated *ssbΔ35* cells did not lead to SOS induction (not shown). Conversely to what is observed at non permissive temperature, this strongly indicates that other members of the SSB_Cter_ interactome are needed for generating and/or stabilizing the ssDNA template for the loading of RecA upon MMC treatment. Obvious candidates are the RecQ and RecJ proteins, a helicase/exonuclease couple known to generate the ssDNA from damaged DNA or inactivated replication forks, onto which the RecFOR machinery mediates RecA delivery (reviewed in [2]). The SOS response defect in *ssbΔ35* cells has an important bearing on the results of a recent study on RecA localization in *B. subtilis* cells. GFP-RecA focus formation on the genome provoked by DNA damaging agents (including MMC) was shown to depend on replisome activity although RecA does not appear to be pre-recruited at active forks [34]. Our results suggest a mechanism to explain this conditional RecA localization. We propose that the active fork itself has the potential to load RecA directly onto ssDNA already available or produced *de novo* via the SSB_Cter_.

### Defining the role of the SSB_Cter_ in the rescue of arrested forks

**Figure 7:**
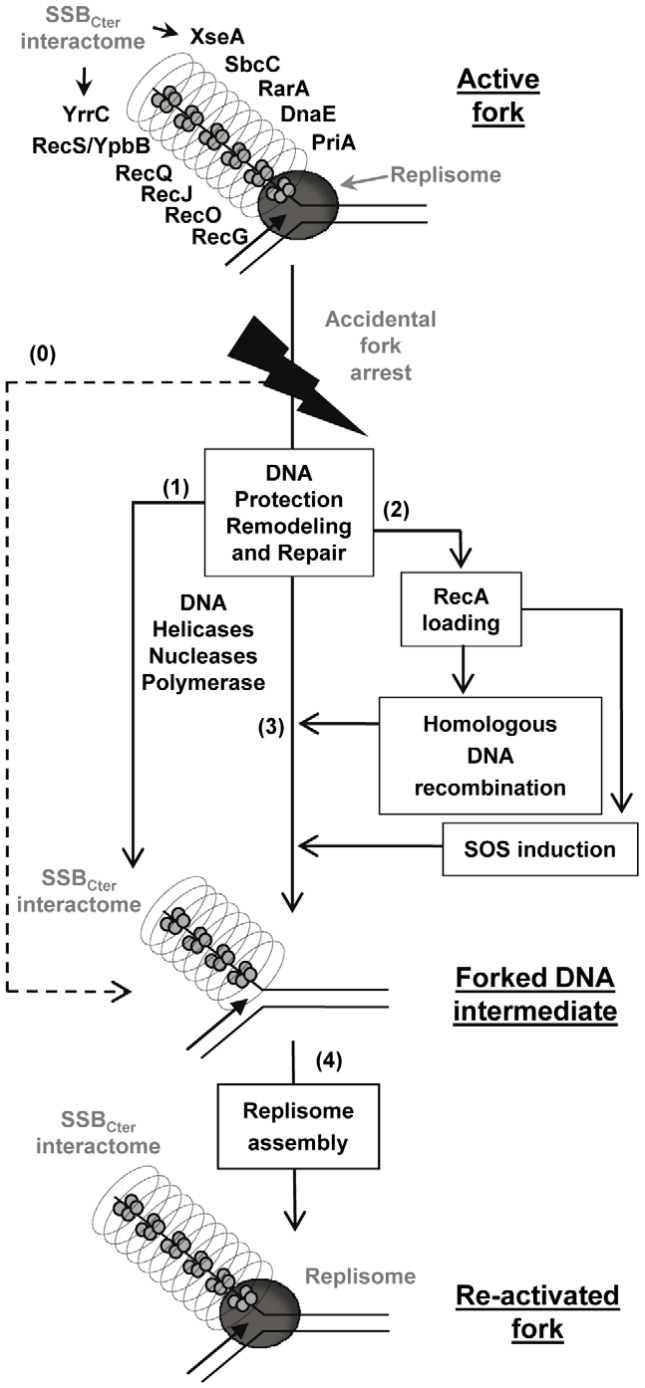
Model of the SSB_Cter_ role in repair of damaged chromosomal DNA replication forks. Replication fork re-activation is depicted as a two-stages process. The first aims at restoring the structural integrity of the inactivated fork (steps 1, 2 and 3). The second consists in replisome re-assembly on the repaired fork (step 4). The active fork is pictured with the replisome (drawn as a grey circle) at the intersection of the parental and replica DNA duplexes. The SSB-coated ssDNA strand corresponds to the lagging-strand template, which is surrounded by the SSB_Cter_ interactome shown as a cylinder. The dotted arrow (step 0) represents replisome disassembly as a consequence of fork arrest, leading directly to the forked DNA substrate of replication restart proteins. The solid arrows (steps 1, 2, 3, 4) represent all the possible routes of fork processing that could be undertaken by SSB partners to attempt the repair and restart of the arrested fork (see text). These routes are not necessarily sequential or interdependent. In this representation, the SSB_Cter_ pre-selects specific DNA effectors, which, once anchored at the fork, would act in a stochastic manner and depending on whether their substrate is present. Thus, the fork might be restarted (step 4), while a DNA lesion is left behind to be solved later (step 1, 2, 3).

Together, these results support a model in which the SSB_Cter_ interactome associated with active forks provides a series of solutions for promoting their restart upon accidental blockage (Figure 7), as well as for dealing with errors left behind the passage of the fork. A key step is replisome assembly on the branched DNA backbone of the fork. The PriA protein and its interaction with the SSB_Cter_ are central to this event [8]. This could be the only response necessary if arrest is due to replisome dismantling. In more complex situations, other actions aim at protecting and/or clearing the fork, *via* individual or concerted actions of the many members of SSB_Cter_ interactome. DNA repair is obviously crucial. This could be handled either immediately by the proteins already present, or delayed *via* RecA loading that could then act in two ways. RecA may reconstruct the fork by homologous recombination, and may induce the SOS response to provide more effectors for DNA repair. Amongst the new effectors coming into play are the error-prone DNA polymerases. Interestingly, it has been shown that the recruitment of *E. coli* PolV at the 3′end of a DNA gap flanked by RecA filaments is increased by an interaction with the *_Ec_*SSB_Cter_ [51]. A distinct class of repair pathways not drawn in the model of Figure 7, are those acting on lesions caused by the replisome but not accompanied by fork arrest. ssDNA gaps are prominent examples of such lesions. In such cases, ssDNA gaps are expected to remain coated by several copies of SSB still interacting with or attracting the proteins that will promote repair.

In conclusion, the SSB_Cter_ emerges as a general maintenance pivot of bacterial genome integrity. Long stretches of ssDNA are intimately associated with the functioning of active bacterial forks. These form primary targets of SSB in living cells and, consequently, of its interactome. One consequence is that replisomes of chromosomal forks are escorted throughout their progression along the bacterial genome (generally for more than 2 Mbp per fork, as in the case of *E. coli* and *B. subtilis* model bacteria). In addition and in a reciprocal way, the forks behave as vehicles for many DNA repair proteins, providing also a convenient way to scan DNA integrity during genome duplication.

## Materials and Methods

### Bacterial strains and plasmids

*B. subtilis* strains used in this study, all based on the 168 or L1430 derivatives, are listed in Table S2 along with the strategies used for their construction. They were propagated in LB medium supplemented, unless otherwise indicated, with appropriate antibiotics (erythromycin, 0.6 µg/ml; spectinomycin, 60 µg/ml; chloramphenicol, 5 µg/ml; tetracycline, 15 µg/ml, phleomycin, 2 µg/ml). *ssb3^+^*, *ssbΔ35*, *ssbΔ*6 and all strains carrying a gene tagged with the SPA motif at its locus were maintained with IPTG (1 mM). Expression of a gene under the P_xyl_ promoter was achieved by adding 0.2% of D-xylose to the medium. All new chromosomal structures were verified by PCR using appropriate pairs of primers. In case of insertions at the *amyE* locus, these were also verified by the loss of amylase activity on starch containing media plates.

*E. coli* strains used were MiT898 [15] for plasmid constructions and ER2566 from NEB, Rosetta (DE3 pLys) or BL21-Gold (DE3) from Novagen for protein expression and purification.

All plasmids used in this study are listed in Table S3. Details of their construction are presented in Text S1.

### Microscopy and analysis of the localization patterns

Microscopy analyses were done as described previously [8]. Cells, grown at 30°C until mid-exponential growth phase in LB medium supplemented with appropriate antibiotics and 0.2% D-xylose, were examined with a Leica DMRA2 microscope equipped with a ×100 magnification oil-immersion objective and a COOLSNAP HQ camera (Roper Scientific, USA). Images were captured and processed with METAMORPH V7.5r5.

### Tap-tag of SPA fusions in *B. subtilis*

Except for SSB, the SPA purification tag [52] was joined to the 3′ end of each gene candidate at its original locus. Tandem affininity purifications were performed as previously described [8] with slight modifications, which are detailed in Text S1.

### Purification of the proteins produced in *E. coli*

Purification procedures of all the proteins produced in *E. coli* used in this study are described in Text S1.

### Pull-down assays on magnetic ssDNA beads coated by SSB proteins

25 µl of Dynabeads M-280 streptavidin per assay (Invitrogen) were incubated 15 min at 4°C in 20 mM Tris-HCl pH 7.5, 2 M NaCl with 50 pmol of a 65-mer oligonucleotide 5′-CGTCGTTTTACAACGTCGTGACTGGGAAAACCCTGGCGTTACCCAACTTAATCGCCTTGCAGCA-3′ biotinilated (with biotin TEG; Genecust). Beads were washed with 200 µl of the same buffer, resuspended in 200 µl of buffer B (20 mM Tris-HCl pH 7.5; 200 mM NaCl) supplemented with 80 pmol of purified SSB or SSBΔ6 and incubated at 20°C under agitation (800 rpm) in a 96 wells plate in a Thermomixer (Eppendorf). Beads were washed in 200 µl of buffer B and resuspended in 200 µl of the same buffer supplemented with various quantities of purified DnaE, PcrA, RecO, RecG, RecQ, or RarA proteins as indicated in the figures. After 30 min of incubation at 800 rpm and 20°C, beads were washed in 200 µl of buffer B, drained and resuspended in 10 µl of SDS-PAGE loading buffer. The proteins were separated on 14% SDS-PAGE and revealed by Coomassie blue staining.

### Cell survival assays

Spot assays were used to measure the viability of *B. subtilis* strains used in this study. O/N cultures, incubated at 30°C or 37°C, as indicated in the figure legend, were diluted in fresh LB medium at the same temperature supplemented as indicated in the figure legend with erythromycin, IPTG and with or without D-xylose. At mid-log phase (A_650nm_≈0.3), 10 µl of 10-fold dilutions (10^0^ to 10^−5^ in Figure 5 and 10^−1^ to 10^−6^ in Figure 6) were spotted on LB agar plates containing the same antibiotics and inducers as those used in the liquid culture. Plates were then incubated O/N at different temperatures (as indicated in the figure legends). In UV resistance assays, plates were exposed to UV irradiation at the indicated doses prior to O/N incubation at 37°C. Colonies were counted after 24 or 48 hours of growth (depending on their growth rate and/or the incubation temperature). Cell survival was expressed as the ratios of the CFU (Colony Forming Units) of UV-treated to untreated cells or of CFU obtained at the tested temperature to CFU obtained at 37°C (Figure 5) or 30°C (Figure 6) for each strain.

### SOS response assays

O/N cultures of strains containing the P_lexA_:*lacZ* cassette at *amyE* were propagated at 37°C in LB medium supplemented with erythromycin, spectinomycin and IPTG. Cells in exponential phase (A_650nm_≈0.03), obtained by inoculating O/N cultures in fresh LB medium supplemented with erythromycin and IPTG, were treated or not with 40 ng/ml MMC to induce or not the SOS response respectively. Sample of ∼0.5 ml per unit of A_650nm_ were taken from cultures every 30 min and treated as described previously [53] for the determination of β-galactosidase activity, expressed in nmol of ONP produced per minute and per mg of protein.

### Western blot analysis

Whole protein extracts and western blot analysis were done as previously described [8] with slight modifications as reported in Text S1.

## Supporting Information

Figure S1 (A, B) The *ypbB*-*recS* locus organization is conserved in the *Bacillales* and *Lactobacillales* Orders. A search for *B. subtilis ypbB*-*recS* locus type organisation was done on all sequenced bacterial genomes using the Region Genome Comparison tool from JVCI (59: a minimum of 40% similarity was used). 53 *ypbB*/*recS* locus type organisations were identified (panel A, a total of 526 sequenced genomes was used). The number of sequenced genomes containing a *ypbB-recS* locus type is given and compared to the total number of sequenced genomes in different groups of bacteria. Examples of four *ypbB*/*recS* loci are given in panel B in three *Bacillales* (*B. subtilis 168*, *Staphylococcus aureus subsp aureus MRSA252* and *Listeria monocytogenes EGD*), and one *Lactobacillales* (*Lactobacillus plantarum WCFS1*). Coordinates of these genes on the chromosome are indicated. (C) YpbB displays significant sequence similarities with the C-terminal part of some extended RecQ proteins. A search for proteins with homology to YpbB in bacteria using the sequence of *B. subtilis* YpbB and the NCBI Blast tool 59 has led to the identification of YpbB proteins in *Firmicutes* (all associated with a RecS homologous protein). Surprisingly, sequence similarity was found between YpbB and the C-terminal part (approximately the 300 last amino-acids) of proteins annotated as RecQ in different bacteria. All the sequences of these proteins are longer (more than 700 residues) than canonical RecQ from *B. subtilis* (590 residues) or *E. coli* (610 residues). An example of this similarity is shown in the panel C by the alignment of YpbB with the C-terminal domain of RecQ2 of *Bacillus cereus* ATCC10987 (Bce 2842).
(0.49 MB TIF)

Figure S2 RecS and YpbB form a complex able to interact with SSB *in vitro*. Purified RecS (4 µM) or YpbB/RecS (4 µM) were mixed on ice with SSB (16 µM in panel C, or 40 µM in panel E) and loaded onto a gel-filtration column. Final concentrations for proteins correspond to their monomeric forms. Fractions (0.5 ml, numbered on the top of the gels) were analyzed by 12.5% SDS-PAGE and Coomassie blue staining. Molecular masses in kDa of standard proteins used to calibrate the sizing column are indicated below the last gel. RecS does not stably interact with SSB, as judged by its identical elution from the column when loaded alone or mixed with SSB. (compare panels A and C). By contrast, RecS associates into a complex with YpbB, as judged by a shift in its elution from the column (compare panel A and D). Equivalent amounts of YpbB and RecS appear to be present in the complex. Notably, the apparent molecular mass of RecS is lower than its theorictical mass (43 kDa versus 57 kDa). The same is true for the YpbB/RecS complex, the apparent mass of which is 65 kDa compare with the theoritical mass of 98 kDa if made of one monomer of RecS and of YpbB. These differences argue for a non globular shape of RecS. Finally, the elution of YpbB/RecS complex from the sizing column is further upon mixing with SSB, indicating that the YpbB/RecS complex interacts physically with SSB (compare panels D and E).
(0.95 MB TIF)

Figure S3 The PcrA and DinG DNA helicases fused to the GFP do not form foci on the nucleoid of *B. subtilis* and do not interact directly or indirectly with SSB. (A) GFP and DAPI fluorescent signals in exponentially growing cultures of *B. subtilis* cells carrying GFP fused to the N-terminus of PcrA (on the left) or DinG (on the right) produced by induction of the P_xyl_ promoter. Cells were grown in LB supplemented with 0.2% D-xylose. (B) Isolation and identification of protein complexes containing the PcrA-SPA (on the left) or the DinG-SPA (on the right) protein. Cell extract was prepared and treated as described in Material and Methods. Proteins were analysed by SDS-PAGE and Coomassie blue staining. Visible bands were analyzed by MALDI-TOF mass spectrometry. PcrA* or DinG* indicates PcrA or DinG degradation products respectively. Unannotated bands correspond to proteins that gave neither a spectrum nor a match in the predicted *B. subtilis* proteins database. Contaminants most often recovered by Tap-tag from *B. subtilis* have been indicated in italic, as a matter of distinction with the others considered as specific partners.
(0.63 MB TIF)

Figure S4 A pull-down assay for testing protein interaction with the *B. subtilis* SSB_Cter_ domain. Pull down assays of interaction between purified RecQ (panel A), RecG (panel B), and PcrA (panel C) proteins and SSB or SSBΔ6 bound to 5′ biotinylated ssDNA oligonucleotides linked to magnetic streptavidin beads (see details in Figure 2).
(0.73 MB TIF)

Figure S5 Pull-down assay of interaction between PriA, RecG, DnaE, and SSB or SSBΔ35. Experiments were performed as described in Figure 2 and Figure S4 with purified SSBΔ35 instead of SSBΔ6. In this experiment, 60 pmol of PriA (lanes 1 and 2), RecG (lanes 3 and 4), or DnaE (lanes 5 and 6) (indicated by a black triangle on the 14% SDS-PAGE Coomassie-stained) were added to ssDNA magnetic beads coated by 100 pmol (in tetramer) of SSB (lanes 1, 3 and 5) or SSBΔ35 (lanes 2, 4 and 6).
(0.51 MB TIF)

Figure S6 SSB interacts with RarA. Further validation of SSB_Cter_-dependent interaction between RarA and SSB by gel filtration. RarA (25 µM) and/or SSB or SSBΔ6 (25 µM) were mixed on ice and loaded onto a gel-filtration column. Final concentrations for proteins correspond to their monomeric forms. Fractions (0.5 ml, numbered on the top of the stained gels) were analyzed by 12.5% SDS-PAGE and Coomassie blue staining. Molecular masses of standard proteins are indicated.
(0.37 MB TIF)

Figure S7 Co-localization of YFP-RecO with DnaX-CFP at *B. subtilis* active chromosomal forks. A. YFP (yellow), CFP (blue) and overlay of both fluorescent images in RecO^+^ cells carrying the *YFP-recO* and *dnaX-CFP* constructs. Experiments were done exactly as for co-localization of PriA and DnaX in [8]. Visible CFP and YFP foci are indicated by white triangles. B. Statistical analysis of the co-localization of DnaX-CFP and YFP-RecO. The percentage of co-localized and individual foci of the two fusions have been calculated from 756 scored foci.
(0.61 MB TIF)

Figure S8 SSB_Cter_ deletion induces various morphological defects. (A) Growth kinetics in LB medium supplemented with erythromycin and IPTG at 37°C of the *ssbΔ35* strain (triangles) and *ssb3^+^* control strain (squares). Growth was followed by monitoring Colony Forming Units (CFU)/ml as described in Figure 4A. Error bars indicate the standard deviation from the mean calculated from two independent experiments. (B) The viability defect of the *ssbΔ35* strain is accompanied by cell (B) and/or nucleoid (C) morphological defects throughout the growth period. These defects were observed by phase contrast (black), or by FM4-64 (red) and DAPI staining (blue), which allow visualization of the membrane and the nucleoid, respectively. Morphological types (B) observed during growth of *ssb3^+^* and/or *ssbΔ35* cells are classified into three groups: (i) normal cell morphology (normal), (ii) very long cells with a septation defect generating filaments (filamentous) and (iii) cells lacking nucleoid for which membranes were clearly visible (anucleated, indicated by a white arrow) or almost undetectable (phantom) after FM4-64 staining and observation with an epifluorescence microscope. Nucleoid morphology (C) observed in *ssb3^+^* and *ssbΔ35* cells are classified (i) normal, (ii) small, (iii) guillotine (when bisected by a septum; white arrow). For each strain, at least 150 cells for each time point were observed and classified into the previously described groups.
(0.64 MB TIF)

Figure S9 *ssbΔ6* cells suffer the same growth defects and sensitivity to UV and MMC as *ssbΔ35* cells. (A, B) Growth kinetics of *ssbΔ35* (triangles), *ssbΔ6* (circles) and *ssb3^+^* (squares) strains in LB supplemented with erythromycin and IPTG at 37°C. Growth was followed by monitoring A_650nm_ (A) and Colony Forming Unit/ml (CFU, (B)) as a function of time. (C) UV sensitivity of *ssb3^+^* (squares), *ssbΔ35* (triangles) and *ssbΔ6* (circles) cells grown in LB at 37°C with erythromycin and IPTG. (D) MMC sensitivity of *ssb3^+^* (squares), *ssbΔ35* (triangles) and *ssbΔ6* (circles) cells grown in LB at 37°C with erythromycin and IPTG. For all panels, an average of at least three independent experiments is reported. Error bars indicate the standard deviation from the mean calculated from all independent experiments.
(0.23 MB TIF)

Table S1 Average number of GFP foci per nucleoid of *B. subtilis* SSB proteins partners fused to GFP in *ssb3^+^* or C-terminal mutant of *ssb*. GFP fusion proteins were visualized by GFP fluorescence and nucleoids by DAPI staining. The average number of foci per nucleoid is presented in the right hand column.
(0.06 MB DOC)

Table S2 *B. subtilis* strains used during this work. a. *ssb3+*, *ssbΔ35* and *ssbΔ6* encode wild-type and C-terminal truncated forms of SSB, respectively. In these three strains, the essential *rpsR* gene, which is located immediately after *ssb*, is placed under the control of a P_spac_ promoter. b. These strains were constructed by transformation of competent FLB22 or FLB23 or FLB25 cells with the genomic DNA of the corresponding 168 *amyE::*P_xyl_ *:gfp-gene* strain. c. These strains were constructed by transformation of competent FLB22 or FLB23 or FLB25 cells with pSG1729 or pSG1154 derivatives. d. SPA tagged genes are under the control of their natural promoter, and the downstream *orfs* are under the control of the IPTG inducible P_spac_ promoter. e. These strains were constructed by transformation of competent 168 cells with JJS100 genomic DNA or pFL43. f. FLB52 cells were transformed with FLB22, FLB23 or MAS617 genomic DNA. g. FLB53, FLB54 and FLB55 cells were transformed with FLB56 genomic DNA. h. These strains were obtained by transformation of the corresponding parental strains with genomic DNA from the HVS567 strain. i.j. These strains were constructed by transformation of 168 *Δupp, dinR3* cells with genomic DNA of FLB22 or FLB23 cells (i) then by plasmid pFL43 (j). k. These strains were constructed by transformation of FLB53 or FLB54 cells by JJS100 genomic DNA. l. These strains were constructed by transformation of the corresponding parental strains by genomic DNA of FLB22 or FLB23 cells. m. The 168-derivative strain carrying the *dnaX-cfp* construct was kindly provided by P. Lewis (University of Newcastle, Callaghan, Australia). n. This strain was constructed by transformation of PPBJ417 competent cells by pSMG205. Θ indicates insertion/duplication of the *recO* gene at its chromosomal locus, generated by plasmid integration.
(0.17 MB DOC)

Table S3 Plasmids used and constructed during this work. a: antibiotic resistance markers Ap: ampicilin; Ery: erythromycin; Spec: spectynomycin; Phleo: phleomycin; Kan: kanamycin.
(0.11 MB DOC)

Text S1 Supplementary materials and methods.
(0.06 MB DOC)
