# Supplementary figures and images for: The C-Terminal Domain of the Bacterial SSB Protein Acts as a DNA Maintenance Hub at Active Chromosome Replication Forks
Source: PLoS Genet. 2010 Dec 9;6(12):e1001238. doi: 10.1371/journal.pgen.1001238 (PMC3000357; doi:10.1371/journal.pgen.1001238)

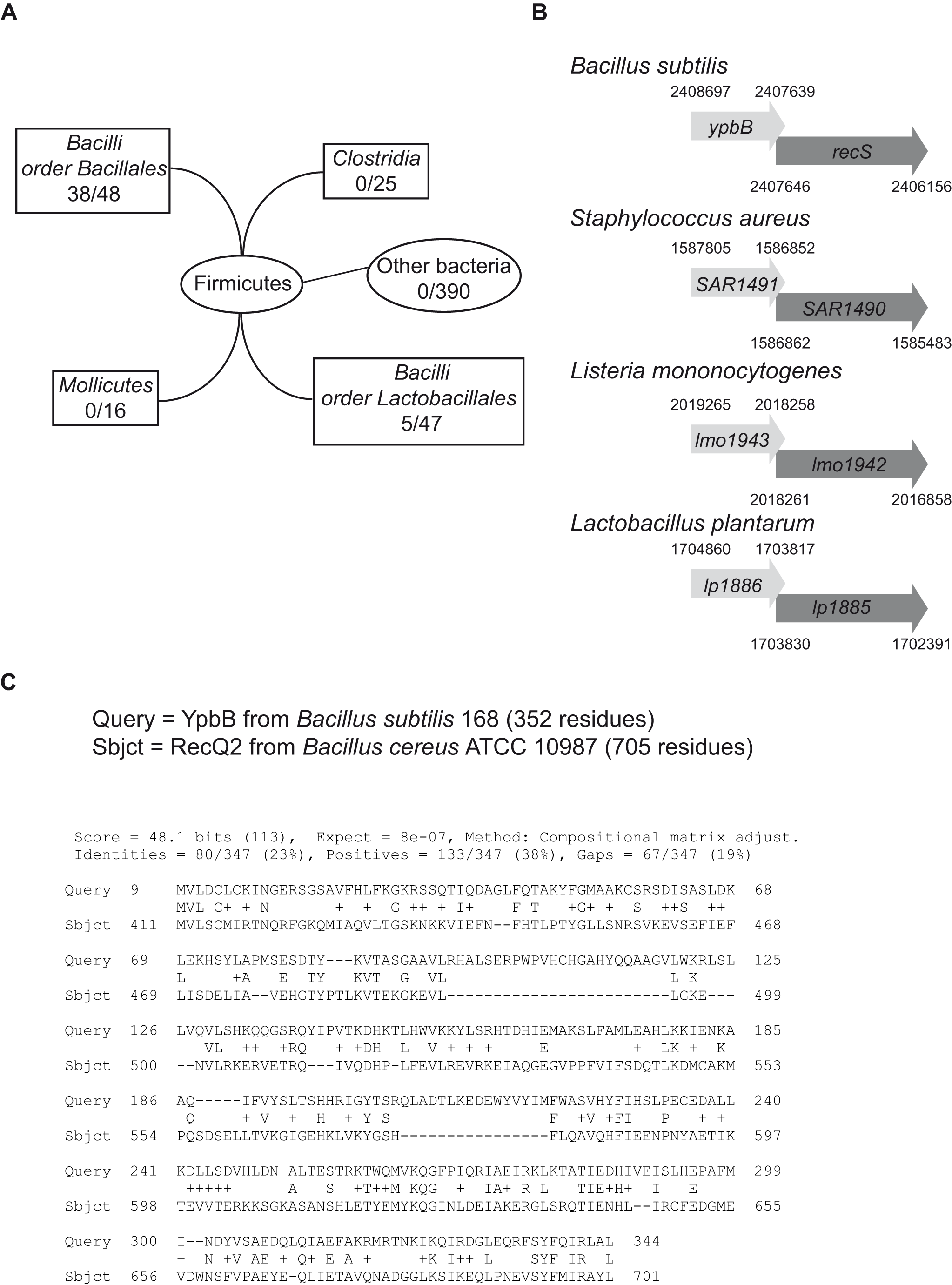

Supplement: Figure S1 — (A, B) The ypbB-recS locus organization is conserved in the Bacillales and Lactobacillales Orders. A search for B. subtilis ypbB-recS locus type organisation was done on all sequenced bacterial genomes using the Region Genome Comparison tool from JVCI (59: a minimum of 40% similarity was used). 53 ypbB/recS locus type organisations were identified (panel A, a total of 526 sequenced genomes was used). The number of sequenced genomes containing a ypbB-recS locus type is given and compared to the total number of sequenced genomes in different groups of bacteria. Examples of four ypbB/recS loci are given in panel B in three Bacillales (B. subtilis 168, Staphylococcus aureus subsp aureus MRSA252 and Listeria monocytogenes EGD), and one Lactobacillales (Lactobacillus plantarum WCFS1). Coordinates of these genes on the chromosome are indicated. (C) YpbB displays significant sequence similarities with the C-terminal part of some extended RecQ proteins. A search for proteins with homology to YpbB in bacteria using the sequence of B. subtilis YpbB and the NCBI Blast tool 59 has led to the identification of YpbB proteins in Firmicutes (all associated with a RecS homologous protein). Surprisingly, sequence similarity was found between YpbB and the C-terminal part (approximately the 300 last amino-acids) of proteins annotated as RecQ in different bacteria. All the sequences of these proteins are longer (more than 700 residues) than canonical RecQ from B. subtilis (590 residues) or E. coli (610 residues). An example of this similarity is shown in the panel C by the alignment of YpbB with the C-terminal domain of RecQ2 of Bacillus cereus ATCC10987 (Bce 2842). (0.49 MB TIF) [file pgen.1001238.s001.tif]

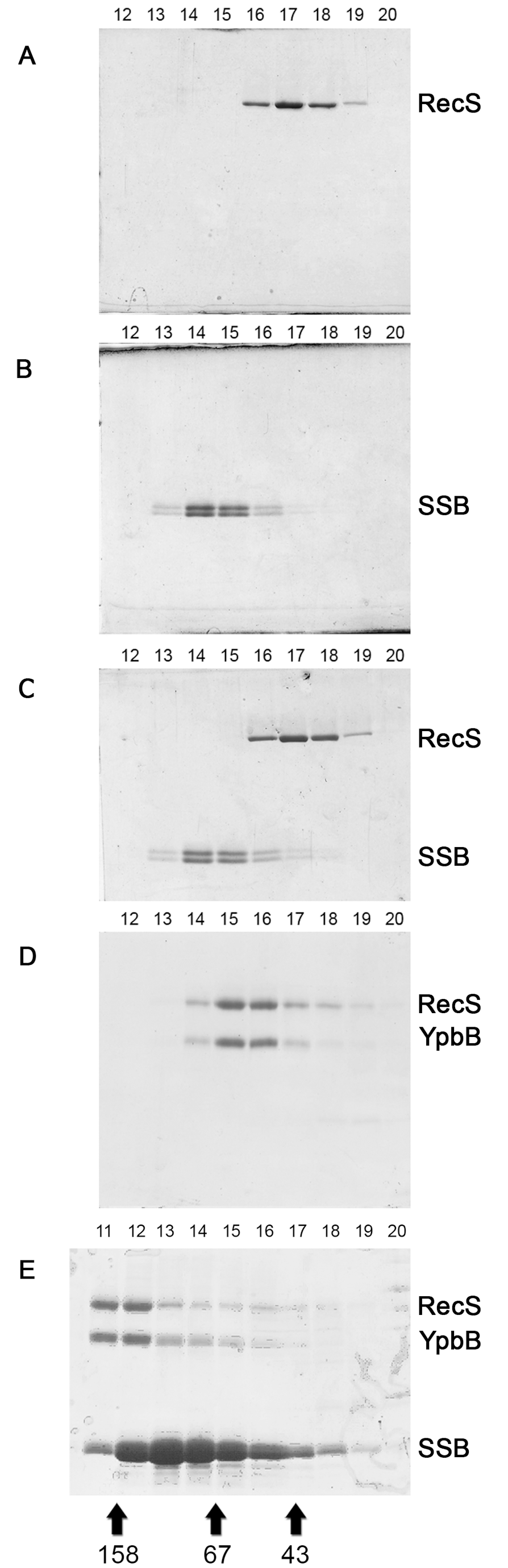

Supplement: Figure S2 — RecS and YpbB form a complex able to interact with SSB in vitro. Purified RecS (4 µM) or YpbB/RecS (4 µM) were mixed on ice with SSB (16 µM in panel C, or 40 µM in panel E) and loaded onto a gel-filtration column. Final concentrations for proteins correspond to their monomeric forms. Fractions (0.5 ml, numbered on the top of the gels) were analyzed by 12.5% SDS-PAGE and Coomassie blue staining. Molecular masses in kDa of standard proteins used to calibrate the sizing column are indicated below the last gel. RecS does not stably interact with SSB, as judged by its identical elution from the column when loaded alone or mixed with SSB. (compare panels A and C). By contrast, RecS associates into a complex with YpbB, as judged by a shift in its elution from the column (compare panel A and D). Equivalent amounts of YpbB and RecS appear to be present in the complex. Notably, the apparent molecular mass of RecS is lower than its theorictical mass (43 kDa versus 57 kDa). The same is true for the YpbB/RecS complex, the apparent mass of which is 65 kDa compare with the theoritical mass of 98 kDa if made of one monomer of RecS and of YpbB. These differences argue for a non globular shape of RecS. Finally, the elution of YpbB/RecS complex from the sizing column is further upon mixing with SSB, indicating that the YpbB/RecS complex interacts physically with SSB (compare panels D and E). (0.95 MB TIF) [file pgen.1001238.s002.tif]

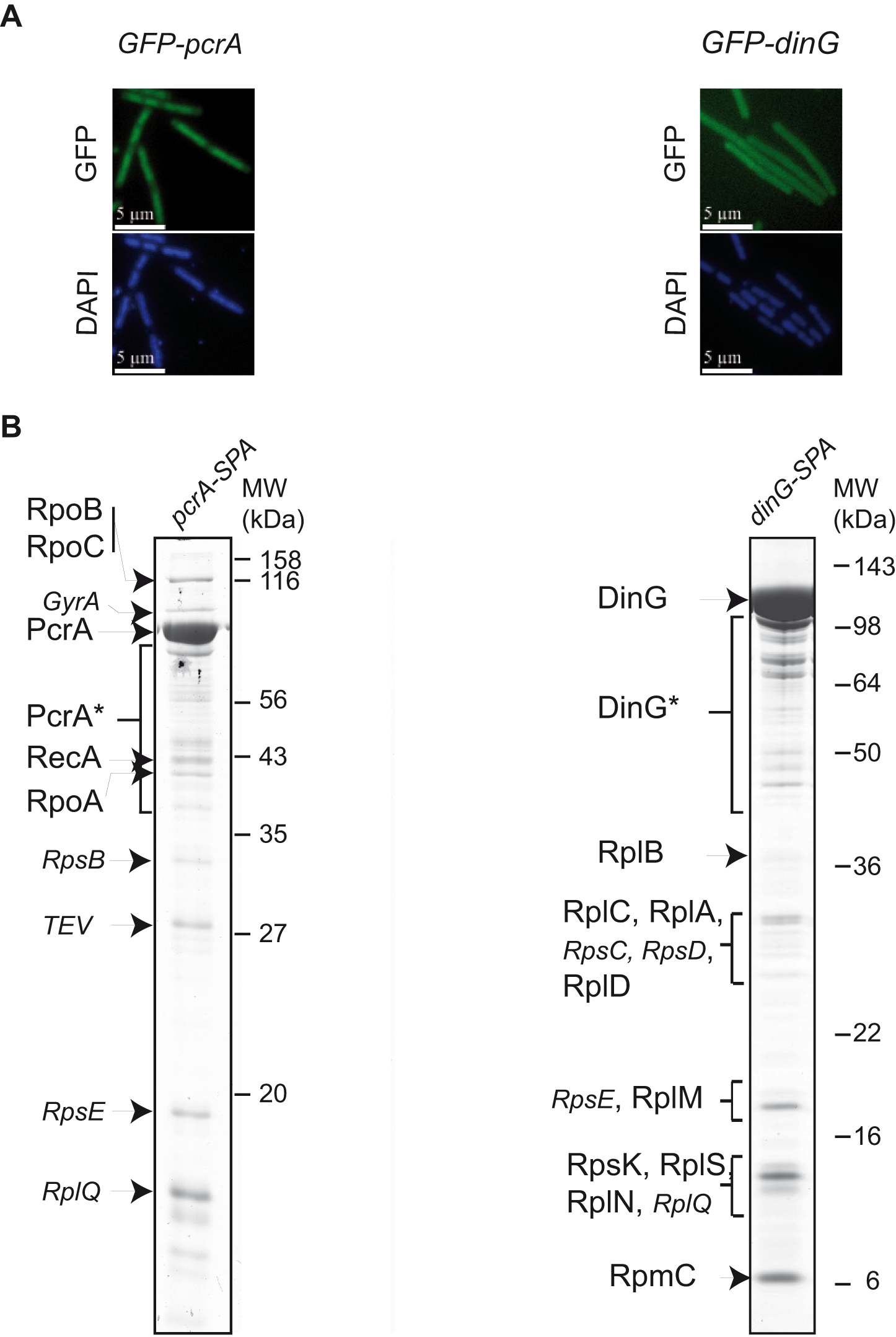

Supplement: Figure S3 — The PcrA and DinG DNA helicases fused to the GFP do not form foci on the nucleoid of B. subtilis and do not interact directly or indirectly with SSB. (A) GFP and DAPI fluorescent signals in exponentially growing cultures of B. subtilis cells carrying GFP fused to the N-terminus of PcrA (on the left) or DinG (on the right) produced by induction of the Pxyl promoter. Cells were grown in LB supplemented with 0.2% D-xylose. (B) Isolation and identification of protein complexes containing the PcrA-SPA (on the left) or the DinG-SPA (on the right) protein. Cell extract was prepared and treated as described in Material and Methods. Proteins were analysed by SDS-PAGE and Coomassie blue staining. Visible bands were analyzed by MALDI-TOF mass spectrometry. PcrA* or DinG* indicates PcrA or DinG degradation products respectively. Unannotated bands correspond to proteins that gave neither a spectrum nor a match in the predicted B. subtilis proteins database. Contaminants most often recovered by Tap-tag from B. subtilis have been indicated in italic, as a matter of distinction with the others considered as specific partners. (0.63 MB TIF) [file pgen.1001238.s003.tif]

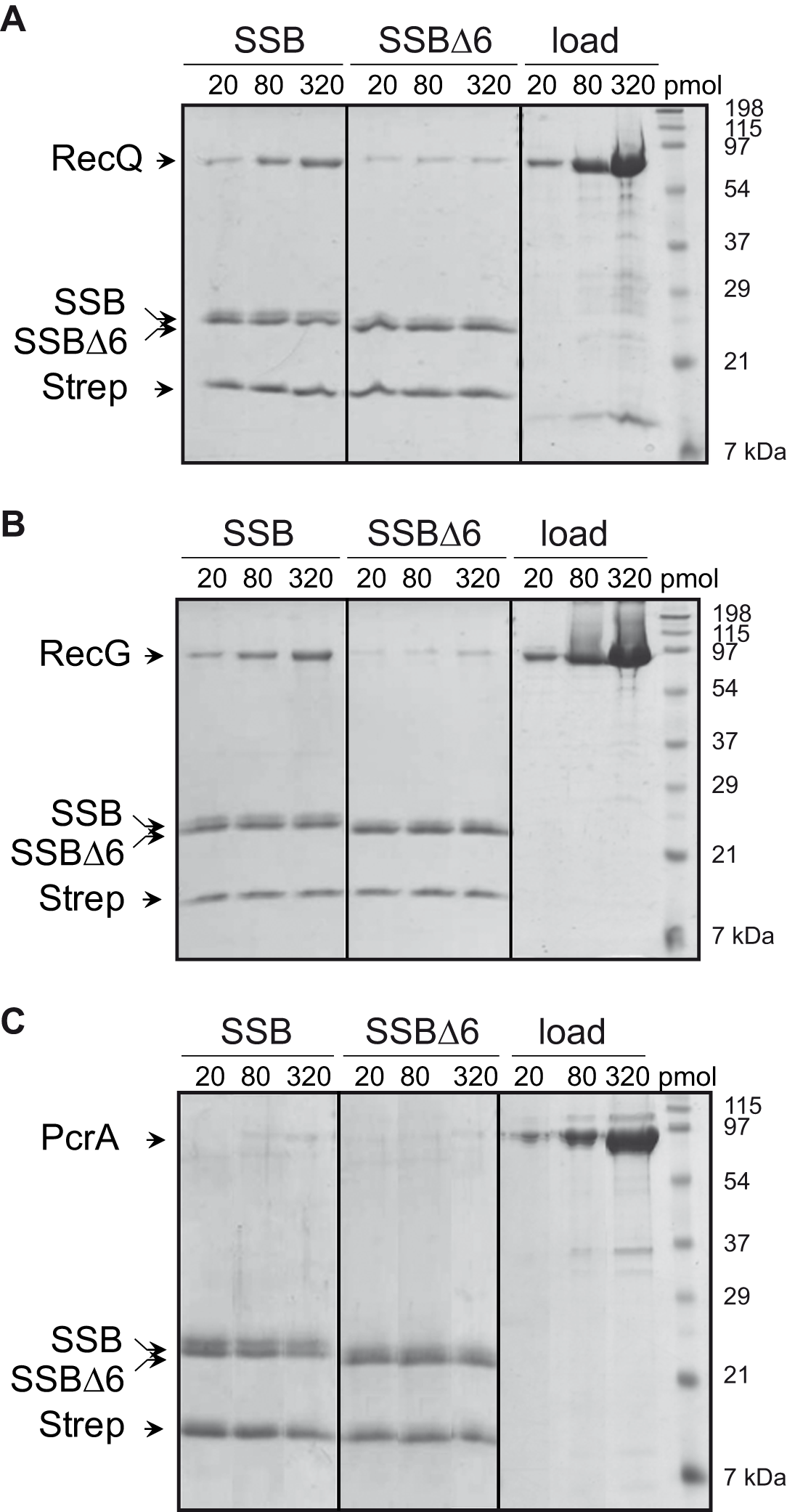

Supplement: Figure S4 — A pull-down assay for testing protein interaction with the B. subtilis SSBCter domain. Pull down assays of interaction between purified RecQ (panel A), RecG (panel B), and PcrA (panel C) proteins and SSB or SSBΔ6 bound to 5′ biotinylated ssDNA oligonucleotides linked to magnetic streptavidin beads (see details in Figure 2). (0.73 MB TIF) [file pgen.1001238.s004.tif]

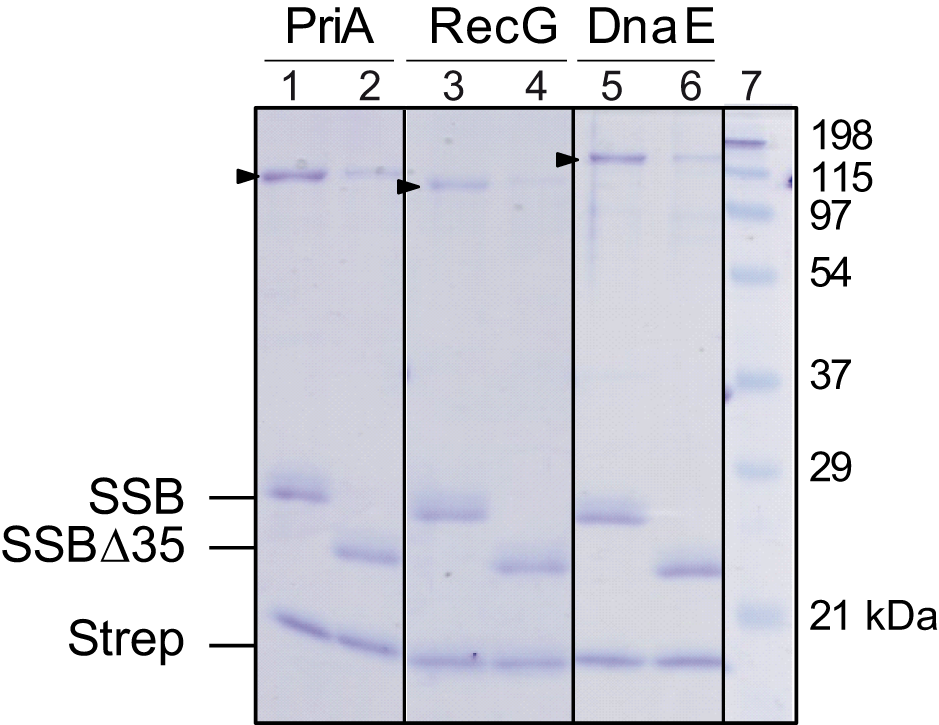

Supplement: Figure S5 — Pull-down assay of interaction between PriA, RecG, DnaE, and SSB or SSBΔ35. Experiments were performed as described in Figure 2 and Figure S4 with purified SSBΔ35 instead of SSBΔ6. In this experiment, 60 pmol of PriA (lanes 1 and 2), RecG (lanes 3 and 4), or DnaE (lanes 5 and 6) (indicated by a black triangle on the 14% SDS-PAGE Coomassie-stained) were added to ssDNA magnetic beads coated by 100 pmol (in tetramer) of SSB (lanes 1, 3 and 5) or SSBΔ35 (lanes 2, 4 and 6). (0.51 MB TIF) [file pgen.1001238.s005.tif]

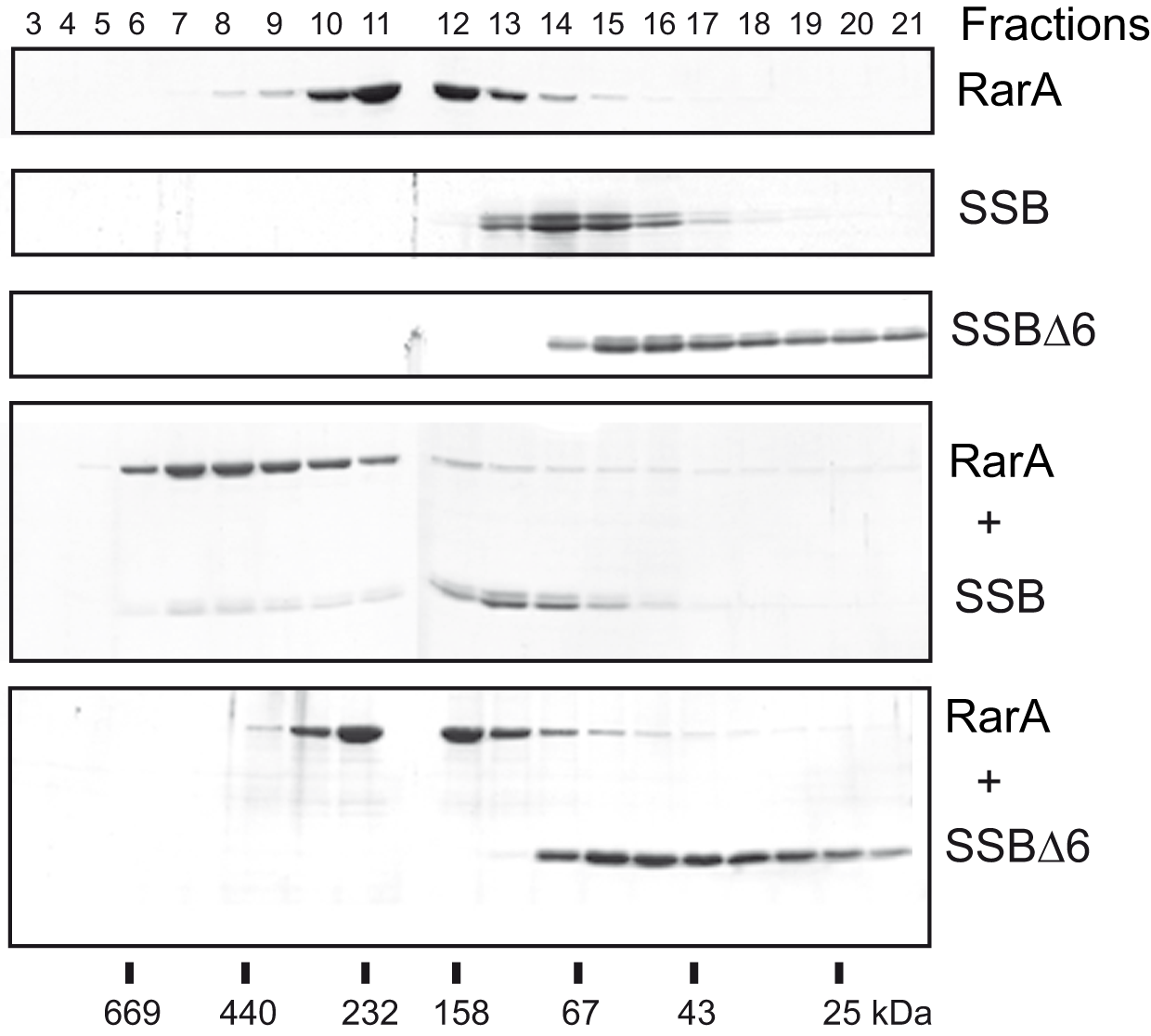

Supplement: Figure S6 — SSB interacts with RarA. Further validation of SSBCter-dependent interaction between RarA and SSB by gel filtration. RarA (25 µM) and/or SSB or SSBΔ6 (25 µM) were mixed on ice and loaded onto a gel-filtration column. Final concentrations for proteins correspond to their monomeric forms. Fractions (0.5 ml, numbered on the top of the stained gels) were analyzed by 12.5% SDS-PAGE and Coomassie blue staining. Molecular masses of standard proteins are indicated. (0.37 MB TIF) [file pgen.1001238.s006.tif]

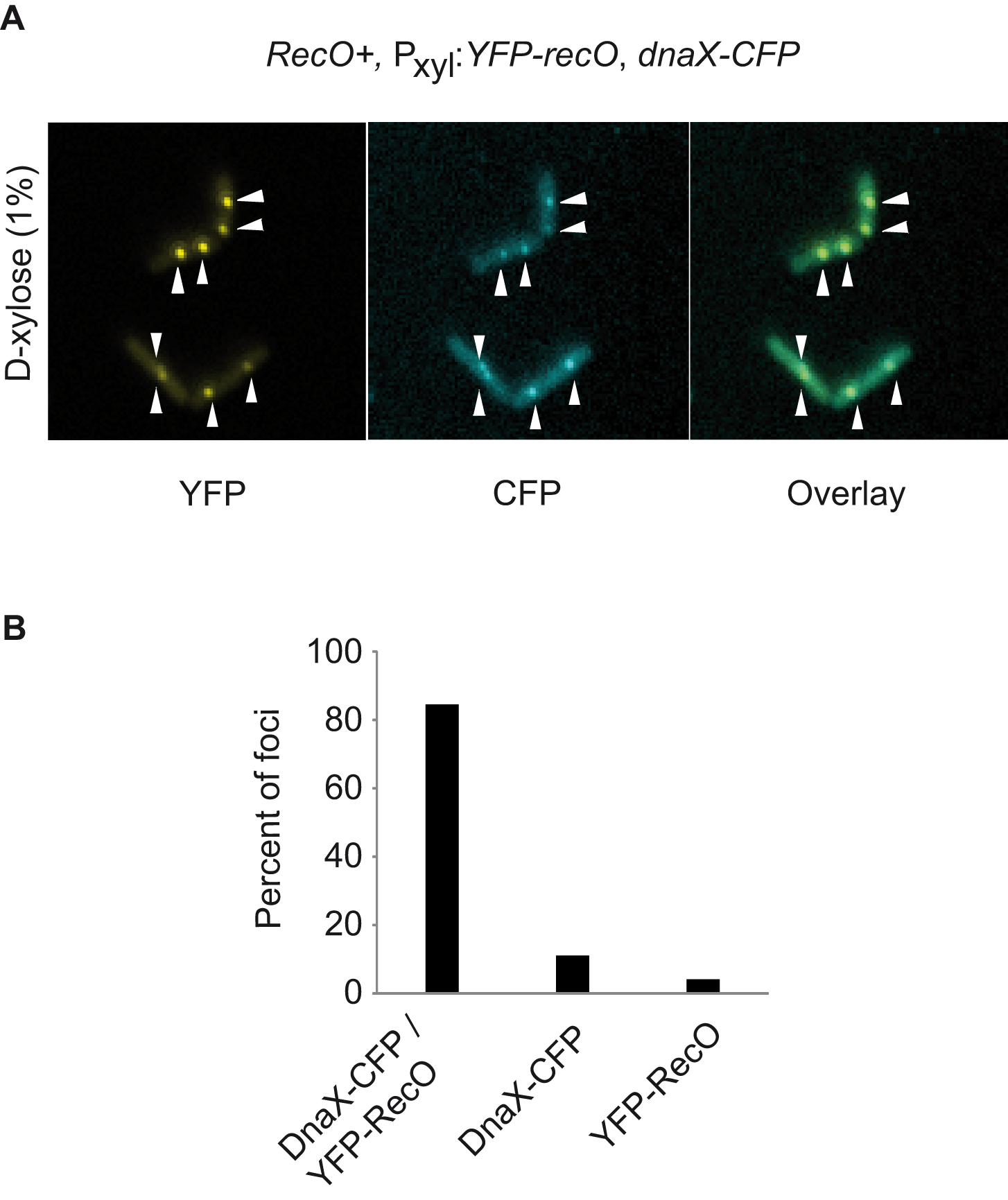

Supplement: Figure S7 — Co-localization of YFP-RecO with DnaX-CFP at B. subtilis active chromosomal forks. A. YFP (yellow), CFP (blue) and overlay of both fluorescent images in RecO+ cells carrying the YFP-recO and dnaX-CFP constructs. Experiments were done exactly as for co-localization of PriA and DnaX in [8]. Visible CFP and YFP foci are indicated by white triangles. B. Statistical analysis of the co-localization of DnaX-CFP and YFP-RecO. The percentage of co-localized and individual foci of the two fusions have been calculated from 756 scored foci. (0.61 MB TIF) [file pgen.1001238.s007.tif]

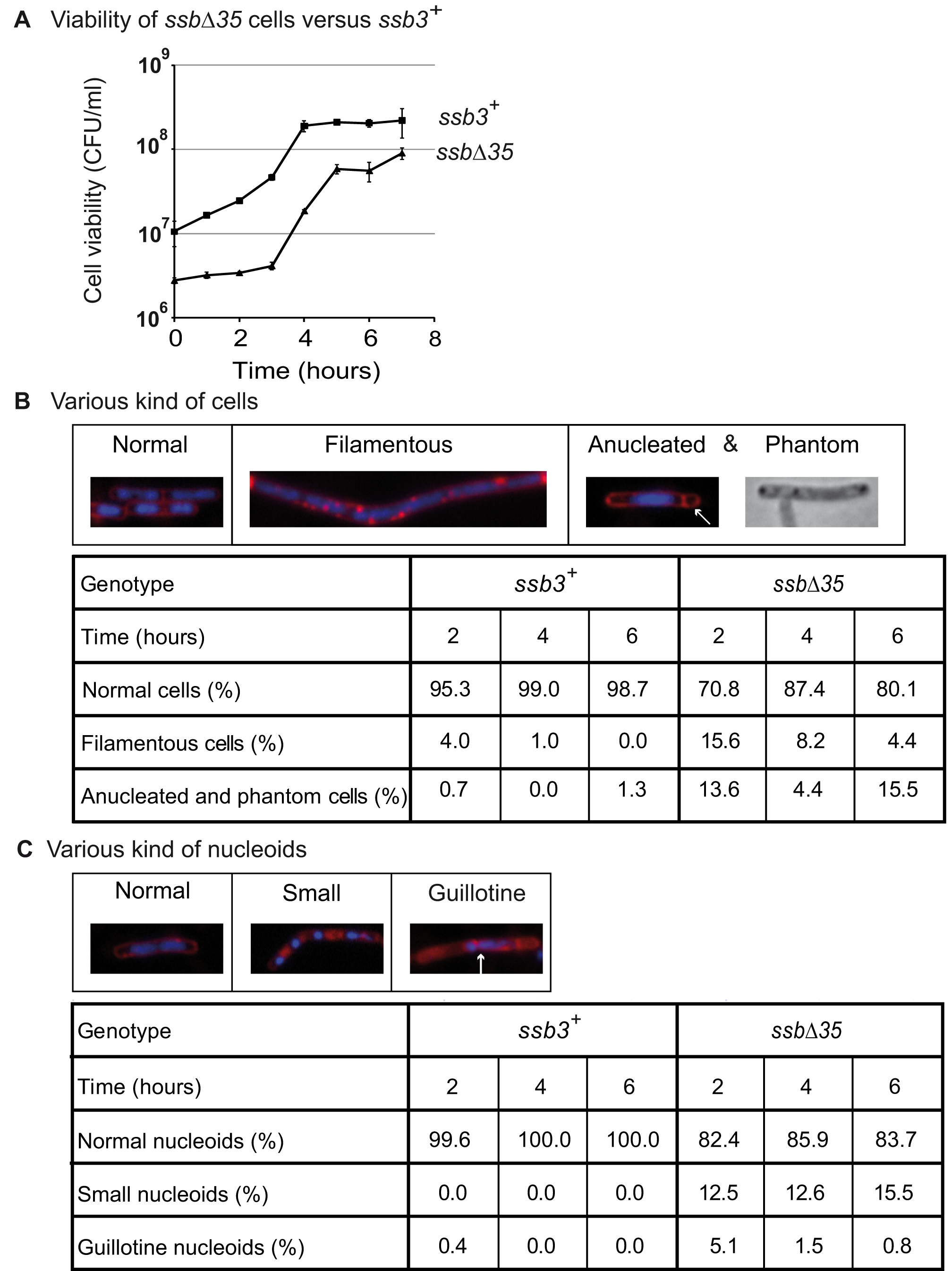

Supplement: Figure S8 — SSBCter deletion induces various morphological defects. (A) Growth kinetics in LB medium supplemented with erythromycin and IPTG at 37°C of the ssbΔ35 strain (triangles) and ssb3+ control strain (squares). Growth was followed by monitoring Colony Forming Units (CFU)/ml as described in Figure 4A. Error bars indicate the standard deviation from the mean calculated from two independent experiments. (B) The viability defect of the ssbΔ35 strain is accompanied by cell (B) and/or nucleoid (C) morphological defects throughout the growth period. These defects were observed by phase contrast (black), or by FM4-64 (red) and DAPI staining (blue), which allow visualization of the membrane and the nucleoid, respectively. Morphological types (B) observed during growth of ssb3+ and/or ssbΔ35 cells are classified into three groups: (i) normal cell morphology (normal), (ii) very long cells with a septation defect generating filaments (filamentous) and (iii) cells lacking nucleoid for which membranes were clearly visible (anucleated, indicated by a white arrow) or almost undetectable (phantom) after FM4-64 staining and observation with an epifluorescence microscope. Nucleoid morphology (C) observed in ssb3+ and ssbΔ35 cells are classified (i) normal, (ii) small, (iii) guillotine (when bisected by a septum; white arrow). For each strain, at least 150 cells for each time point were observed and classified into the previously described groups. (0.64 MB TIF) [file pgen.1001238.s008.tif]

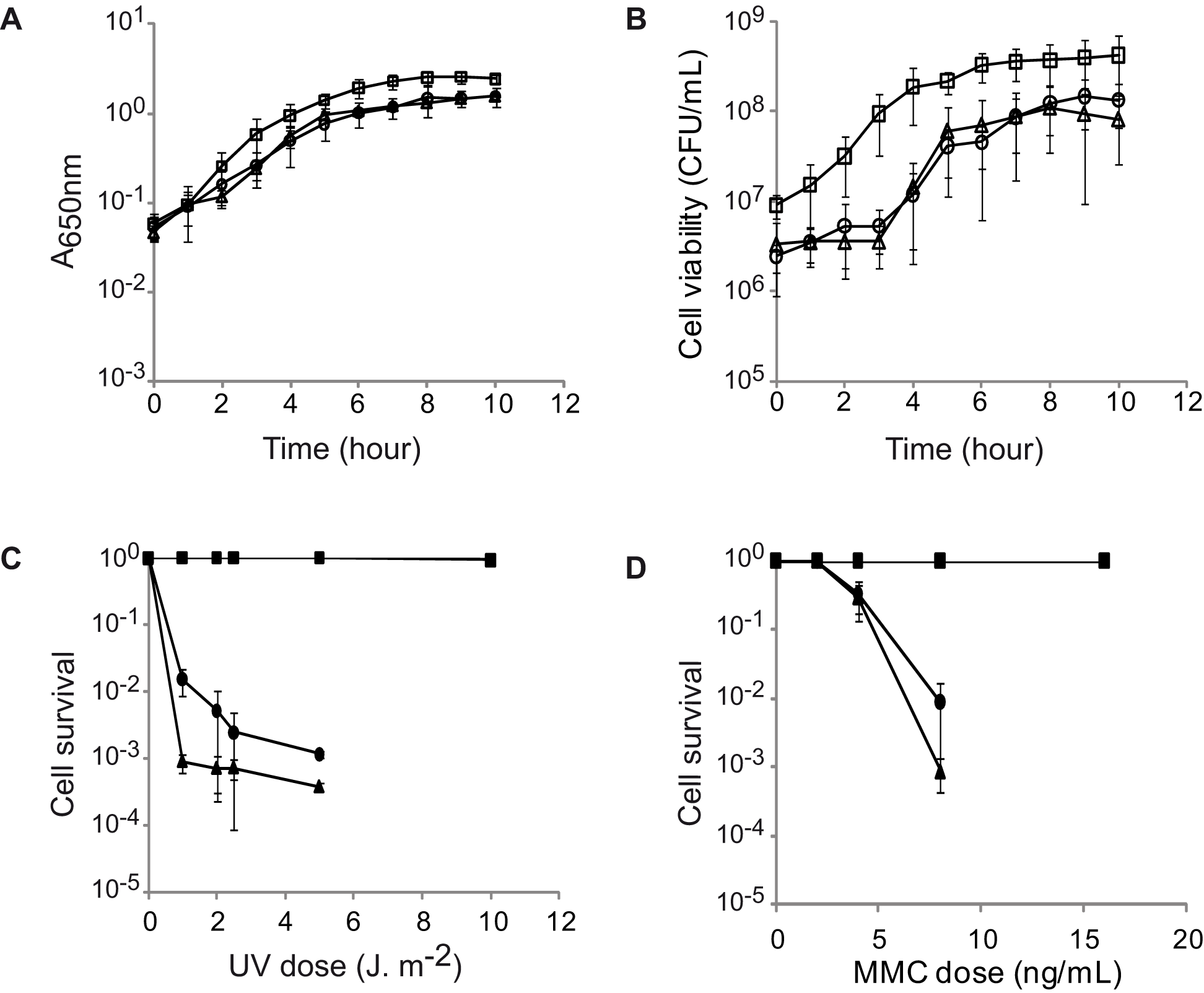

Supplement: Figure S9 — ssbΔ6 cells suffer the same growth defects and sensitivity to UV and MMC as ssbΔ35 cells. (A, B) Growth kinetics of ssbΔ35 (triangles), ssbΔ6 (circles) and ssb3+ (squares) strains in LB supplemented with erythromycin and IPTG at 37°C. Growth was followed by monitoring A650nm (A) and Colony Forming Unit/ml (CFU, (B)) as a function of time. (C) UV sensitivity of ssb3+ (squares), ssbΔ35 (triangles) and ssbΔ6 (circles) cells grown in LB at 37°C with erythromycin and IPTG. (D) MMC sensitivity of ssb3+ (squares), ssbΔ35 (triangles) and ssbΔ6 (circles) cells grown in LB at 37°C with erythromycin and IPTG. For all panels, an average of at least three independent experiments is reported. Error bars indicate the standard deviation from the mean calculated from all independent experiments. (0.23 MB TIF) [file pgen.1001238.s009.tif]
