# Supplementary material for: The C-Terminal Domain of the Bacterial SSB Protein Acts as a DNA Maintenance Hub at Active Chromosome Replication Forks
Source: PLoS Genet. 2010 Dec 9;6(12):e1001238. doi: 10.1371/journal.pgen.1001238 (PMC3000357; doi:10.1371/journal.pgen.1001238)
Supplement: Table S1 — Average number of GFP foci per nucleoid of B. subtilis SSB proteins partners fused to GFP in ssb3+ or C-terminal mutant of ssb. GFP fusion proteins were visualized by GFP fluorescence and nucleoids by DAPI staining. The average number of foci per nucleoid is presented in the right hand column. (0.06 MB DOC) [file pgen.1001238.s010.doc]

| **Strain / *genotype*** | **Nucleoids counted** | **Nucleoids with foci (%)** | **Foci / nucleoid** |
| --- | --- | --- | --- |
| PPBJ334 / *GFP-recS* | 190 | 2.6 | 0.0 |
| PPBJ433 / *GFP-ypbB* | 191 | 8.4 | 0.1 |
| ACB151 / *ssb3+*, *GFP-ypbB-recS* | 165 | 95.8 | 1.8 |
| ACB153 / *ssbΔ35*, *GFP-ypbB-recS* | 226 | 3.1 | 0.0 |
| ACB713 / *ssbΔ6*, *GFP-ypbB-recS* | 232 | 0.0 | 0.0 |
| PPBJ334 / *GFP-pcrA* | 173 | 0.0 | 0.0 |
| FLB42 / *ssb3+*, *dnaE-GFP* | 331 | 85.8 | 1.6 |
| FLB41 / *ssbΔ35*, *dnaE-GFP* | 187 | 0.5 | 0.0 |
| FLB43 / *ssbΔ6*, *dnaE-GFP* | 209 | 0.5 | 0.0 |
| FLB40 / *ssb3+*, *polC-GFP* | 207 | 89.4 | 1.8 |
| FLB39 / *ssbΔ35*, *polC-GFP* | 184 | 82.1 | 1.5 |
| FLB38 / *ssb3+*, *GFP-holA* | 152 | 72.4 | 1.3 |
| FLB37 / *ssbΔ35*, *GFP-holA* | 126 | 76.2 | 1.2 |
| ACB126 / *ssb3+*, *GFP-holB* | 214 | 65.9 | 1.1 |
| ACB125 / *ssbΔ35*, *GFP-holB* | 184 | 71.2 | 1.1 |
| FLB36 / *ssb3+*, *GFP-dnaN* | 204 | 99.5 | 2.1 |
| FLB35 / *ssbΔ35*, *GFP-dnaN* | 211 | 94.3 | 1.8 |
| ACB37 / *ssb3+*, *GFP-dnaC* | 188 | 96.3 | 1.8 |
| ACB25 / *ssbΔ35*, *GFP-dnaC* | 130 | 73.9 | 1.2 |
| FLB32 / *ssb3+*, *GFP-sbcC* | 232 | 68.5 | 0.9 |
| FLB31 / *ssbΔ35*, *GFP-sbcC* | 196 | 2.0 | 0.0 |
| FLB34 / *ssb3+*, *GFP-yabA* | 177 | 90.4 | 1.3 |
| FLB33 / *ssbΔ35*, *GFP-yabA* | 113 | 86.7 | 1.3 |
| PPBJ456 / *ssb3+*, *GFP-rarA* | 257 | 99.6 | 2.6 |
| PPBJ459 / *ssbΔ35*, *GFP-rarA* | 181 | 2.8 | 0.0 |
| FLB45 / *ssbΔ6*, *GFP-rarA* | 199 | 9.0 | 0.1 |
| PPBJ445 / *ssb3+*, *GFP-recJ* | 222 | 91.0 | 1.9 |
| PPBJ447 / *ssbΔ35*, *GFP-recJ* | 205 | 1.5 | 0.0 |
| ACB123 / *ssb3+*, *GFP-recO* | 121 | 99.2 | 2.5 |
| ACB124 / *ssbΔ35*, *GFP-recO* | 113 | 0.0 | 0.0 |
| FLB44 / *ssbΔ6*, *GFP-recO* | 191 | 2.6 | 0.0 |
| PPBJ463 / *ssb3+*, *GFP-xseA* | 264 | 77.3 | 1.9 |
| PPBJ466 / *ssbΔ35*, *GFP-xseA* | 124 | 2.4 | 0.0 |
| PPBJ457 / *ssb3+*, *GFP-yrrC* | 230 | 96.5 | 2.1 |
| PPBJ460 / *ssbΔ35*, *GFP-yrrC* | 185 | 0.5 | 0.0 |

**Table S1 : Average number of GFP foci per nucleoid of *B. subtilis* SSB proteins partners fused to GFP in *ssb3^+^* or C-terminal mutant of *ssb*.**

GFP fusion proteins were visualized by GFP fluorescence and nucleoids by DAPI staining. The average number of foci per nucleoid is presented in the right hand column.
