# Supplementary material for: The C-Terminal Domain of the Bacterial SSB Protein Acts as a DNA Maintenance Hub at Active Chromosome Replication Forks
Source: PLoS Genet. 2010 Dec 9;6(12):e1001238. doi: 10.1371/journal.pgen.1001238 (PMC3000357; doi:10.1371/journal.pgen.1001238)
Supplement: Table S2 — B. subtilis strains used during this work. a. ssb3+, ssbΔ35 and ssbΔ6 encode wild-type and C-terminal truncated forms of SSB, respectively. In these three strains, the essential rpsR gene, which is located immediately after ssb, is placed under the control of a Pspac promoter. b. These strains were constructed by transformation of competent FLB22 or FLB23 or FLB25 cells with the genomic DNA of the corresponding 168 amyE::Pxyl :gfp-gene strain. c. These strains were constructed by transformation of competent FLB22 or FLB23 or FLB25 cells with pSG1729 or pSG1154 derivatives. d. SPA tagged genes are under the control of their natural promoter, and the downstream orfs are under the control of the IPTG inducible Pspac promoter. e. These strains were constructed by transformation of competent 168 cells with JJS100 genomic DNA or pFL43. f. FLB52 cells were transformed with FLB22, FLB23 or MAS617 genomic DNA. g. FLB53, FLB54 and FLB55 cells were transformed with FLB56 genomic DNA. h. These strains were obtained by transformation of the corresponding parental strains with genomic DNA from the HVS567 strain. i.j. These strains were constructed by transformation of 168 Δupp, dinR3 cells with genomic DNA of FLB22 or FLB23 cells (i) then by plasmid pFL43 (j). k. These strains were constructed by transformation of FLB53 or FLB54 cells by JJS100 genomic DNA. l. These strains were constructed by transformation of the corresponding parental strains by genomic DNA of FLB22 or FLB23 cells. m. The 168-derivative strain carrying the dnaX-cfp construct was kindly provided by P. Lewis (University of Newcastle, Callaghan, Australia). n. This strain was constructed by transformation of PPBJ417 competent cells by pSMG205. Θ indicates insertion/duplication of the recO gene at its chromosomal locus, generated by plasmid integration. (0.17 MB DOC) [file pgen.1001238.s011.doc]

| *B. subtilis* strains | Relevant genotype | Reference, source |
| --- | --- | --- |
| 168 | *trpC2* | laboratory clone |
| FLB22^a^ | *trpC2, ssbΔ35* | [1] |
| FLB23^a^ | *trpC2, ssb3+* | [1] |
| FLB25^a^ | *trpC2, ssbΔ6* | this work |
| 861 | *trpC2,*  *amyE*::P_xyl_:*dnaE-GFP* | [2] |
| FLB42^b^ | *trpC2, ssb3+, amyE*::P_xyl_:*dnaE-GFP* | this work |
| FLB41^b^ | *trpC2, ssbΔ35, amyE*::P_xyl_:*dnaE-GFP* | this work |
| FLB43^b^ | *trpC2, ssbΔ6,*  *amyE*::P_xyl_:*dnaE-GFP* | this work |
| FLB40^c^ | *trpC2, ssb3+, amyE*::P_xyl_:*polC-GFP* | this work |
| FLB39^c^ | *trpC2, ssbΔ35,*  *amyE*::P_xyl_:*polC-GFP* | this work |
| 441 | *trpC2,*  *amyE*::P_xyl_:*GFP-dnaN* | [3] |
| FLB36^b^ | *trpC2, ssb3+, amyE*::P_xyl_:*GFP-dnaN* | this work |
| FLB35^b^ | *trpC2, ssbΔ35,*  *amyE*::P_xyl_:*GFP-dnaN* | this work |
| FLB38^c^ | *trpC2, ssb3+, amyE*::P_xyl_:*GFP-holA* | this work |
| FLB37^c^ | *trpC2, ssbΔ35,*  *amyE*::P_xyl_:*GFP-holA* | this work |
| ACB126^c^ | *trpC2, ssb3+, amyE*::P_xyl_:*GFP-holB* | this work |
| ACB125^c^ | *trpC2, ssbΔ35,*  *amyE*::P_xyl_:*GFP-holB* | this work |
| ACB37^c^ | *trpC2, ssb3+, amyE*::P_xyl_:*GFP-dnaC* | this work |
| ACB25^c^ | *trpC2, ssbΔ35,*  *amyE*::P_xyl_:*GFP-dnaC* | this work |
| 1724H | *trpC2,*  *amyE*::P_xyl_:*GFP-sbcC* | [3] |
| FLB32^b^ | *trpC2, ssb3+, amyE*::P_xyl_:*GFP-sbcC* | this work |
| FLB31^b^ | *trpC2, ssbΔ35, amyE*::P_xyl_:*GFP-sbcC* | this work |
| FLB34^c^ | *trpC2, ssb3+, amyE*::P_xyl_:*GFP-yabA* | this work |
| FLB33^c^ | *trpC2, ssbΔ35, amyE*::P_xyl_:*GFP-yabA* | this work |
| PPBJ456^c^ | *trpC2, ssb3+, amyE*::P_xyl_:*GFP-rarA* | this work |
| PPBJ459^c^ | *trpC2, ssbΔ35, amyE*::P_xyl_:*GFP-rarA* | this work |
| FLB45^c^ | *trpC2, ssbΔ6,*  *amyE*::P_xyl_:*GFP-rarA* | this work |
| PPBJ445^c^ | *trpC2, ssb3+, amyE*::P_xyl_:*GFP-recJ* | this work |
| PPBJ447^c^ | *trpC2, ssbΔ35, amyE*::P_xyl_:*GFP-recJ* | this work |
| ACB123^c^ | *trpC2, ssb3+, amyE*::P_xyl_:*GFP-recO* | this work |
| ACB124^c^ | *trpC2, ssbΔ35, amyE*::P_xyl_:*GFP-recO* | this work |
| FLB44^c^ | *trpC2, ssbΔ6,*  *amyE*::P_xyl_:*GFP-recO* | this work |
| PPBJ463^c^ | *trpC2, ssb3+, amyE*::P_xyl_:*xseA-GFP* | this work |
| PPBJ466^c^ | *trpC2, ssbΔ35, amyE*::P_xyl_:*xseA-GFP* | this work |
| PPBJ457^c^ | *trpC2, ssb3+, amyE*::P_xyl_:*GFP-yrrC* | this work |
| PPBJ460^c^ | *trpC2, ssbΔ35, amyE*::P_xyl_:*GFP-yrrC* | this work |
| PPBJ332 | *trpC2,*  *amyE*::P_xyl_:*GFP-pcrA* | this work |
| PPBJ334 | *trpC2,*  *amyE*::P_xyl_:*GFP-recS* | this work |
| PPBJ433 | *trpC2,*  *amyE*::P_xyl_:*GFP-ypbB* | this work |
| PPBJ431 | *trpC2,*  *amyE*::P_xyl_:*GFP-ypbB-recS* | this work |
| PPBJ320 | *trpC2, amyE*::P_xyl_:*GFP-recG* | this work |
| ACB151^b^ | *trpC2, ssb3+, amyE*::P_xyl_:*gfp-ypbB-recS* | this work |
| ACB153^b^ | *trpC2, ssbΔ35, amyE*::P_xyl_:*GFP-ypbB-recS* | this work |
| ACB713^b^ | *trpC2, ssbΔ6, amyE*::P_xyl_:*GFP-ypbB-recS* | this work |
| FLB50 | *trpC2, amyE*::P_xyl_:*ssb-SPA* | this work |
| ACB373 | *trpC2, amyE*::P_xyl_:*ssb* | this work |
| FLB5^d^ | *trpC2, dnaE-SPA* | this work |
| FLB9^d^ | *trpC2, recQ-SPA* | [2] |
| FLB16^d^ | *trpC2, recS-SPA* | this work |
| FLB46^d^ | *trpC2, pcrA-SPA* | this work |
| FLB47^d^ | *trpC2,* *recJ-SPA* | this work |
| FLB48^d^ | *trpC2,* *recO-SPA* | this work |
| FLB49^d^ | *trpC2, rarA-SPA* | this work |
| JJS100 | TF8a Δupp *amyE::*P_lexA_*:lacZ* | [4] |
| FLB52^e^ | *trpC2, amyE::*P_lexA_*:lacZ* | this work |
| FLB53^f^ | *trpC2, amyE::*P_lexA_*:lacZ, ssb3+* | this work |
| FLB54^f^ | *trpC2, amyE::*P_lexA_*:lacZ,*  *ssbΔ35* | this work |
| MAS617 | *trpC2,* *recO1::cm* | [5] |
| FLB55^f^ | *trpC2,*  *amyE::*P_lexA_*:lacZ, recO1::cm* | this work |
| FLB56^e^ | *trpC2,*  *lacA::* P_xyl_*:recO-SPA* | this work |
| FLB57^g^ | *trpC2,*  *amyE::*P_lexA_*:lacZ, ssb3+, lacA::* P_xyl_*:recO-SPA* | this work |
| FLB58^g^ | *trpC2,*  *amyE::*P_lexA_*:lacZ, ssbΔ35,*  *lacA::* P_xyl_*:recO-SPA* | this work |
| FLB59^g^ | *trpC2,*  *amyE::*P_lexA_*:lacZ, recO1::cm,* *lacA::* P_xyl_*:recO-SPA* | this work |
| HVS567 | *trpC2,* *recA::tet* | [6] |
| FLB60^h^ | *trpC2, ssb3+,* *recA::tet* | this work |
| FLB61^h^ | *trpC2, ssbΔ35,* *recA::tet* | this work |
| FLB62^h^ | *trpC2, amyE::*P_lexA_*:lacZ, ssb3+, lacA::* P_xyl_*:recO-SPA, recA::tet* | this work |
| FLB63^h^ | *trpC2, amyE::*P_lexA_*:lacZ, ssbΔ35, lacA::* P_xyl_*:recO-SPA, recA::tet* | this work |
| 168 *Δupp, dinR3* | *trpC2, Δupp dinR3* | [7] |
| FLB64^i^ | *trpC2, Δupp dinR3, ssb3+* | this work |
| FLB65^i^ | *trpC2, Δupp dinR3, ssbΔ35* | this work |
| FLB66^j^ | *trpC2, Δupp dinR3, ssb3+, lacA::* P_xyl_*:recO-SPA* | this work |
| FLB67^j^ | *trpC2, Δupp dinR3, ssbΔ35, lacA::* P_xyl_*:recO-SPA* | this work |
| FLB68^k^ | *trpC2, Δupp dinR3, ssb3+, amyE::*P_lexA_*:lacZ* | this work |
| FLB69^k^ | *trpC2, Δupp dinR3, ssbΔ35, amyE::*P_lexA_*:lacZ* | this work |
| L1430 | *metC, ilvA, lys21* | [8] |
| L1434 | *metC, lys21, dnaD23* | [8] |
| L1437 | *metC, lys21, dnaN5* | [8] |
| L1438 | *metC, ilvA, dnaX51* | [8] |
| L1435 | *metC, ilvA, dnaG20* | [8] |
| ACB513^l^ | *metC, ilvA, lys21, ssb3+* | this work |
| ACB515^l^ | *metC, lys21, dnaD23, ssb3+* | this work |
| ACB517^l^ | *metC, lys21, dnaN5, ssb3+* | this work |
| ACB519^l^ | *metC, ilvA, dnaX51, ssb3+* | this work |
| ACB521^l^ | *metC, ilvA, dnaG20, ssb3+* | this work |
| ACB527^l^ | *metC, ilvA, lys21, ssbΔ35* | this work |
| ACB529^l^ | *metC, lys21, dnaD23, ssbΔ35* | this work |
| ACB531^l^ | *metC, lys21, dnaN5, ssbΔ35* | this work |
| ACB533^l^ | *metC, ilvA, dnaX51, ssbΔ35* | this work |
| ACB535^l^ | *metC, ilvA, dnaG20, ssbΔ35* | this work |
| PPBJ417^m^ | *trpC2, dnaX-CFP* | P. Lewis |
| ppBJ423^n^ | *trpC2, dnaX-CFP,* *recO* Θ P_xyl_*:YFP-recO* | this work |

**Table S2: *B. subtilis* strains used during this work.**

**a.** *ssb3+*, *ssbΔ35* and *ssbΔ6* encode wild-type and C-terminal truncated forms of SSB, respectively. In these three strains, the essential *rpsR* gene, which is located immediately after *ssb*, is placed under the control of a P_spac_ promoter. **b.** These strains were constructed by transformation of competent FLB22 or FLB23 or FLB25 cells with the genomic DNA of the corresponding 168 *amyE::*P_xyl_*:gfp-gene* strain. **c**. These strains were constructed by transformation of competent FLB22 or FLB23 or FLB25 cells with pSG1729 or pSG1154 derivatives. **d.** SPA tagged genes are under the control of their natural promoter, and the downstream *orfs* are under the control of the IPTG inducible P_spac_ promoter. **e**. These strains were constructed by transformation of competent 168 cells with JJS100 genomic DNA or pFL43. **f.** FLB52 cells were transformed with FLB22, FLB23 or MAS617 genomic DNA. **g.** FLB53, FLB54 and FLB55 cells were transformed with FLB56 genomic DNA. **h.** These strains were obtained by transformation of the corresponding parental strains with genomic DNA from the HVS567 strain. **i.j.** These strains were constructed by transformation of 168 *Δupp, dinR3* cells with genomic DNA of FLB22 or FLB23 cells (i) then by plasmid pFL43 (j). **k.** These strains were constructed by transformation of FLB53 or FLB54 cells by JJS100 genomic DNA. **l.** These strains were constructed by transformation of the corresponding parental strains by genomic DNA of FLB22 or FLB23 cells. **m.** The 168-derivative strain carrying the *dnaX-cfp* construct was kindly provided by P. Lewis (University of Newcastle, Callaghan, Australia). **n.** This strain was constructed by transformation of PPBJ417 competent cells by pSMG205. Θ indicates insertion/duplication of the *recO* gene at its chromosomal locus, generated by plasmid integration.

**References**

1. Lecointe F, Serena C, Velten M, Costes A, McGovern S, et al. (2007) Anticipating chromosomal replication fork arrest: SSB targets repair DNA helicases to active forks. Embo J 26: 4239-4251.

2. Dervyn E, Suski C, Daniel R, Bruand C, Chapuis J, et al. (2001) Two essential DNA polymerases at the bacterial replication fork. Science 294: 1716-1719.

3. Meile JC, Wu LJ, Ehrlich SD, Errington J, Noirot P (2006) Systematic localisation of proteins fused to the green fluorescent protein in *Bacillus subtilis*: identification of new proteins at the DNA replication factory. Proteomics 6: 2135-2146.

4. Duigou S, Ehrlich SD, Noirot P, Noirot-Gros MF (2004) Distinctive genetic features exhibited by the Y-family DNA polymerases in *Bacillus subtilis*. Mol Microbiol 54: 439-451.

5. Petit MA, Ehrlich D (2002) Essential bacterial helicases that counteract the toxicity of recombination proteins. Embo J 21: 3137-3147.

6. Chedin F, Dervyn E, Dervyn R, Ehrlich SD, Noirot P (1994) Frequency of deletion formation decreases exponentially with distance between short direct repeats. Mol Microbiol 12: 561-569.

7. Fabret C, Ehrlich SD, Noirot P (2002) A new mutation delivery system for genome-scale approaches in *Bacillus subtilis*. Mol Microbiol 46: 25-36.

8. Mauel C, Karamata D (1984) Prophage induction in thermosensitive DNA mutants of *Bacillus subtilis*. Mol Gen Genet 194: 451-456.
