# Supplementary material for: The C-Terminal Domain of the Bacterial SSB Protein Acts as a DNA Maintenance Hub at Active Chromosome Replication Forks
Source: PLoS Genet. 2010 Dec 9;6(12):e1001238. doi: 10.1371/journal.pgen.1001238 (PMC3000357; doi:10.1371/journal.pgen.1001238)
Supplement: Table S3 — Plasmids used and constructed during this work. a: antibiotic resistance markers Ap: ampicilin; Ery: erythromycin; Spec: spectynomycin; Phleo: phleomycin; Kan: kanamycin. (0.11 MB DOC) [file pgen.1001238.s012.doc]

|  | **I. Plasmids used for integration at specific loci of the *B. subtilis* genome.** | | | |
| --- | --- | --- | --- | --- |
| **Name** | **origin and use** | **Resistance marker^a^** | | **Ref.** |
|  |  | **In *E. coli*** | **In *B. subtilis*** |  |
| pSG1729 | ColEI derivative designed to insert *GFP* fusions at the 5’of a gene of interest at *amyE* under the P_xyl_ promoter | Ap^R^ | Spec^R^ | [1] |
| pSG1154 | ColEI derivative designed to insert *GFP* fusions at the 3’ of a gene of interest at *amyE* under the P_xyl_ promoter | Ap^R^ | Spec^R^ | [1] |
| pSMG104 | PSG1729 derivative carrying the *GFP -recG* construct | Ap^R^ | Spec^R^ | [2] |
| pSMG106 | PSG1729 derivative carrying the *GFP -dnaC* construct | Ap^R^ | Spec^R^ | This work |
| pSMG110 | PSG1729 derivative carrying the *GFP -pcrA* construct | Ap^R^ | Spec^R^ | This work |
| pSMG111 | PSG1729 derivative carrying the *GFP -recS* construct | Ap^R^ | Spec^R^ | This work |
| pSMG114 | PSG1729 derivative carrying the *GFP - recO* construct | Ap^R^ | Spec^R^ | This work |
| pSMG115 | PSG1729 derivative carrying the *GFP - holB* construct | Ap^R^ | Spec^R^ | This work |
| PSMG119 | PSG1729 derivative carrying the *GFP -ypbB-recS* construct | Ap^R^ | Spec^R^ | This work |
| PSMG120 | PSG1729 derivative carrying the *GFP -ypbB* construct | Ap^R^ | Spec^R^ | This work |
| PSMG134 | PSG1729 derivative carrying the *GFP -recJ* construct | Ap^R^ | Spec^R^ | This work |
| PSMG135 | PSG1729 derivative carrying the *GFP -rarA* construct | Ap^R^ | Spec^R^ | This work |
| PSMG137 | PSG1729 derivative carrying the *GFP -yrrC* construct | Ap^R^ | Spec^R^ | This work |
| PSMG143 | PSG1154 derivative carrying the *xseA*- *GFP* construct | Ap^R^ | Spec^R^ | This work |
| pFL30 | PSG1729 derivative carrying the *GFP -holA* construct | Ap^R^ | Spec^R^ | This work |
| pFL31 | PSG1154 derivative carrying the *polC*- *GFP* construct | Ap^R^ | Spec^R^ | This work |
| pSG1729-*yabA* | PSG1729 derivative carrying the *GFP -yabA* construct | Ap^R^ | Spec^R^ | [3] |
| pMUTIN-SPA | plasmid designed to insert the SPA tag at a specific loci in the *B. subtilis* chromosome | Ap^R^ | Ery^R^ | [2] |
| pFL6 | pMUTIN-SPA derivative designed to fuse the SPA tag at the 3’ end of *DnaE* | Ap^R^ | Ery^R^ | This work |
| pFL17 | pMUTIN-SPA derivative designed to fuse the SPA tag at the 3’ end of *recS* | Ap^R^ | Ery^R^ | This work |
| pFL26 | pMUTIN-SPA derivative designed to construct the *ssbΔ6* strain | Ap^R^ | Ery^R^ | This work |
| pFL32 | pMUTIN-SPA derivative designed to fuse the SPA tag at the 3’ end of *pcrA* | Ap^R^ | Ery^R^ | This work |
| pFL33 | pMUTIN-SPA derivative designed to fuse the SPA tag at the 3’ end of *recJ* | Ap^R^ | Ery^R^ | This work |
| pFL34 | pMUTIN-SPA derivative designed to fuse the SPA tag at the 3’ end of *recO* | Ap^R^ | Ery^R^ | This work |
| pFL35 | pMUTIN-SPA derivative designed to fuse the SPA tag at the 3’ end of *rarA* | Ap^R^ | Ery^R^ | This work |
| pFL40 | pSG1729 derivative designed to fuse the SPA tag at the 3’end of a gene of interest at *amyE* | Ap^R^ | Spec^R^ | This work |
| pFL41 | pFL40 derivative carrying the *ssb-SPA* construct | Ap^R^ | Spec^R^ | This work |
| pAC19 | pFL40 derivative carrying the *ssb* construct | Ap^R^ | Spec^R^ | This work |
| pAX01 | ColEI derivative designed to express a gene of interest under the P_xyl_ promoter at *lacA* | Ap^R^ | Ery^R^ | [4] |
| Puc19-Phléo | carries a phleomycin resistance gene (*phleo*) under the control of the *B. subtilis* P_sacB_ promoter | Ap^R^, Phleo^R^ |  | [5] |
| pFL42 | Replacement of the *erm* gene of pAX01 by the phleomycin resistance gene under the P_sacB_ promoter of Puc19Phleo | Ap^R^ | Phleo^R^ | This work |
| pFL43 | pFL42 derivative carrying the *recO* construct | Ap^R^ | Phleo^R^ | This work |
| PSG902 | ColEI derivative designed to insert *yfp* fusions at specific loci in the *B. subtilis* chromosome | Ap^R^ | Cm^R^ | P. Lewis |
| pSMG205 | PSG902 derivative carrying the *yfp-recO* construct | Ap^R^ | Cm^R^ | This work |
|  | **II. Plasmids for protein overexpression in *E. coli* and purification.** | | | |
| **Name** | **origin and use** | **Resistance marker in**  ***E. coli ^a^*** | | **Ref.** |
| pTYB1 | Expression vector allowing expression of a gene under the P_T7_ promoter | Ap^R^ | | NEB |
| pSMG69 | pTYB1 derivative carrying the *pcrA* gene without tag/ P_T7_ | Ap^R^ | | This work |
| PSMG146 | pTYB1 derivative carrying the *ssbΔ35* construct/ P_T7_ | Ap^R^ | | This work |
| PSMG148 | pTYB1 derivative carrying the *6His*-*rarA* construct/ P_T7_ | Ap^R^ | | This work |
| pKHS | pET28 derivative designed to polycistronic expression under the P_T7_ promoter and a 3’ tagging or not by a 6-His tag | Kan^R^ | | SQC, unpublished |
| pKHS1 | pKHS derivative carrying the *recO-His* construct | Kan^R^ | | This work |
| pKHS2 | pKHS derivative carrying the *dnaE-His* construct | Kan^R^ | | This work |
| pKHS3 | pKHS derivative carrying the *priA-His* construct | Kan^R^ | | This work |
| pKHS4 | pKHS derivative carrying the *recG-His* construct | Kan^R^ | | This work |
| pKHS5 | pKHS derivative carrying the *recQ-His* construct | Kan^R^ | | This work |
| pKHS6 | pKHS derivative carrying the *ssb* wild type gene/ P_T7_ | Kan^R^ | | This work |
| pKHS7 | pKHS derivative carrying the *ssbΔ6* construct/ P_T7_ | Kan^R^ | | This work |
| pKHS9 | pKHS derivative carrying the *recS-His* construct/ P_T7_ | Kan^R^ | | This work |
| pKHS10 | pKHS derivative carrying the *ypbB-His* construct/ P_T7_ | Kan^R^ | | This work |

**Table S3 : Plasmids used and constructed during this work.**

**a**: antibiotic resistance markers Ap: ampicilin; Ery: erythromycin; Spec: spectynomycin; Phleo: phleomycin; Kan: kanamycin.

**References**

1. Lewis PJ, Marston AL (1999) GFP vectors for controlled expression and dual labelling of protein fusions in *Bacillus subtilis*. Gene 227: 101-110.

2. Lecointe F, Serena C, Velten M, Costes A, McGovern S, et al. (2007) Anticipating chromosomal replication fork arrest: SSB targets repair DNA helicases to active forks. Embo J 26: 4239-4251.

3. Noirot-Gros MF, Velten M, Yoshimura M, McGovern S, Morimoto T, et al. (2006) Functional dissection of YabA, a negative regulator of DNA replication initiation in *Bacillus subtilis*. Proc Natl Acad Sci U S A 103: 2368-2373.

4. Hartl B, Wehrl W, Wiegert T, Homuth G, Schumann W (2001) Development of a new integration site within the *Bacillus subtilis* chromosome and construction of compatible expression cassettes. J Bacteriol 183: 2696-2699.

5. Dervyn E, Suski C, Daniel R, Bruand C, Chapuis J, et al. (2001) Two essential DNA polymerases at the bacterial replication fork. Science 294: 1716-1719.
