# Supplementary material for: The C-Terminal Domain of the Bacterial SSB Protein Acts as a DNA Maintenance Hub at Active Chromosome Replication Forks
Source: PLoS Genet. 2010 Dec 9;6(12):e1001238. doi: 10.1371/journal.pgen.1001238 (PMC3000357; doi:10.1371/journal.pgen.1001238)
Supplement: Text S1 — Supplementary materials and methods. (0.06 MB DOC) [file pgen.1001238.s013.doc]

**Text S1: Supporting Materials and Methods.**

**Plasmid constructions**

All the plasmids used in this study are listed Table S3. The sequences of all the primers used for generating the PCR products used in the plasmids constructions are available upon request.

For the construction of GFP fusions, the coding sequence of each gene of interest was first obtained by PCR from *B. subtilis* genomic DNA using primers designed to add *Apa*I and *Xho*I sites at the ends of the amplicon. The genes were then fused to GFP at their 5’ or 3’ ends by insertion into pSG1729 or pSG1154, respectively. This cloning strategy in pSG1154 led to the addition of five codons upstream of the ATG start codon. The fusions were integrated into the *amyE* locus as described [1].

Plasmid pMUTIN-SPA [2] was used to add the SPA tag coding sequence at the 3’ end of the original chromosomal locus of all genes encoding proteins purified by the Tap-tag procedure. PCR fragments encompassing approximately the last 400 coding nucleotides of each gene were generated using primers that enabled insertion between the *Acc65*I and *Nco*I sites of pMUTIN-SPA.

Plasmid pFL26 (constructed for generating the *ssbΔ6* mutant) was obtained by inserting between the *Acc65*I and *Nco*I sites of pMUTIN-SPA PCR fragments bordered by *Acc65*I and *Nco*I restriction sites and encompassing the internal coding sequence of the *ssb* open reading frame (ORF) from the +14^th^ to the +498^th^ nucleotides (519 nucleotides in the *ssb* wild type ORF) followed by three translational stop codons.

Plasmid pFL40 has been constructed to express a gene of interest from the P_xyl_ promoter at the *B. subtilis amyE* locus, fused or unfused to the SPA sequence at its 3’ end. pFL40 resulted from the exchange of the 1.8 kb *Avr*II/*Xho*I fragment containing the *gfp* gene of pSG1729 by a PCR fragment amplified from pMUTIN-SPA, terminated by sites for AvrII and XhoI and encompassing the SPA tag sequence. Plasmids pFL41 and pAC19 are pFL40 derivatives allowing expression of SSB-SPA and SSB respectively. They were constructed by cloning of a PCR fragment encompassing the *ssb* *gene* without or with three stop codons respectively between the *Avr*II and *Nco*I sites of pFL40.

pFL42, is a derivative of pAX01 [3], which allows the ectopic expression of a gene under the P_xyl_ promoter at the *B. subtilis lacA* locus. To construct pFL42, the *erm* gene (responsible of the erythromycin resistance) of pAX01 was excised by *Not*I/*Sac*II digestion and replaced by a PCR fragment carrying a phleomycin resistance cassette bordered with *Avr*II and *Xho*I restriction sites originating from the pUC19-Phleo plasmid [4]. pFL43 was obtained by insertion at the unique BamHI site of pFL42 of a *Bam*HI DNA fragment encompassing the *recO-SPA* artificial gene (produced by PCR from genomic DNA of the FLB48 strain carrying the *recO-SPA* construct).

Plasmids for gene expression in *E. coli* were constructed as follows. pSMG69 and pSMG146 were constructed by cloning the *pcrA* and *ssbΔ35* coding sequences respectively, as PCR fragments (generated from *B. subtilis* genomic DNA) bordered by *Nde*I and *Nsi*I restriction sites in the pTYB1 plasmid digested by *Nde*I and *Nsi*I. pSMG148 was constructed following the same strategy except that the 5’ primer containing the *Nde*I site also contains six histine codons in front of the beginning of the *rarA* gene. All pKHS derivatives were constructed following an identical strategy: the coding sequence of each gene were amplified using primers flanked by *Eag*I or *Psp*OMI and *Not*I restriction sites and cloned into the pKHS plasmid, a derivative of the pET28 vector (Novagen), cut by the same enzymes. As a result, all proteins expressed from the pKHS are 6His-tagged at their C-terminal end. Plasmids expressing wild-type *B. subtilis* SSB and the mutant SSBΔ6 also derive from the pKHS, except that a stop codon has been added at the 3’ end of the PCR product to express these proteins without any tag.

**Purification of SPA fusions from *B. subtilis***

Overnight (O/N) cultures at 37°C in LB medium with erythromycin and IPTG were diluted 100 fold in 2 liters of the same medium and, in the case of the ectopic expression of SSB or SSB-SPA, supplemented or not with D-xylose as indicated. The cultures were incubated with aeration at 37°C until A_650nm_ ~ 0.5. The cells were harvested, washed in buffer A (10 mM Tris-HCl pH 7.5, 0.15 M NaCl), frozen in liquid nitrogen and kept at –80°C. RecS-SPA and DnaE-SPA were purified as described for RecQ-SPA [2]. PcrA-SPA, RecJ-SPA, RecO-SPA and RarA-SPA were purified using this procedure with the following slight modifications: (i) cell lysis, in which one gram of cells in 2.5 ml of Buffer C (10 mM Tris-HCl pH 7.5, 0.15 M NaCl, 0.2 mM EDTA, 0.1 % Triton X100) supplemented with 1 mg/ml of lysozyme and 50 U of benzonase nuclease (Novagen) was incubated at 37°C for 15 min and rechilled on ice for 10 min; (ii) TEV proteolysis, which was performed with 50U (200U for SSB-SPA) of AcTEV protease (Invitrogen) at 30°C for 1 hour, and (iii) elution from the Anti-FLAG M2 agarose beads (Sigma), which was done directly in TEV buffer. Purified proteins were analysed by 12.5% SDS-PAGE followed by Coomassie blue staining (Bio-Safe Coomassie, BIORAD) and identified by MALDI-TOF mass spectrometry as previously described [2].

**Protein purifications from *E. coli***

Cultures of cells containing pKHS derivatives were grown in 2xYT medium (BIO101Inc.) supplemented with 30 µg/ml kanamycin at 37°C to A_600nm_ = 1, and production of proteins was induced by addition of 0.5 mM IPTG (final concentration). Four hours after induction at 37°C (15°C for YpbB and RecS), cells were harvested by centrifugation, and the pellets resuspended in 40 ml of P Buffer (20 mM Tris-HCl, pH 7.5, 200 mM NaCl) and stored at -20°C. Cells were lysed by sonication and centrifuged at 13000 g for 30 min at 4°C. Supernatants containing the His-tagged proteins were loaded onto a Ni^2+^ affinity column (Ni-NTA agarose, Qiagen) pre-equilibrated in the same buffer. The proteins were eluted with imidazole, and loaded onto a 60x16 Superdex 200 column (GE) equilibrated in P buffer. Wild type SSB was purified as previously described [2] and deletion variants of SSB nearly as the same procedure (exact purification protocols available upon request). The purified proteins were dialysed against P buffer in 50 % glycerol prior to storage at -20°C. 6His-RarA produced from the pSMG148 was purified following the same scheme as described before with minor modifications: cells were grown in LB instead of 2xYT, and treated in 50 mM Tris-HCl, pH 8.0, 500 mM NaCl; the Ni-NTA column was washed by steps with the same buffer supplemented with 20, 40 and 100 mM imidazole. 6His-RarA was eluted with 200 mM imidazole and dialysed against 50 mM Tris-HCl pH 8.0, 0.4 M NaCl, 50 % glycerol and 1 mM DTT prior to storage at -20°C.

YpbB (as a C-terminal fusion with a 6xHis-tag) was found to be insoluble in cell extracts prepared from *E. coli* cells in which it had been overproduced. However, if the lysis is performed together with *E. coli* cells in which RecS had been overexpressed (as a C-terminal fusion with a 6xHis tag), YpbB was found to be soluble and to co-elute with RecS from an Ni-NTA column with 100 mM imidazole. An homogeneous YpbB/RecS species could then be retained on and eluted from an Hitrap-SP column and separated from free unbound RecS. RecS was concentrated on an Hitrap-Q column. The purified and soluble YpbB/RecS and RecS species were then stored at -20°C in 50 mM Tris pH8, 400 mM NaCl, 50% glycerol, 1 mM DTT.

Production of PcrA (plasmid pSMG69) was induced for 1 hr at 30°C with 0.5 mM IPTG added at A_600nm_ = 0.8 in LB supplemented with 100 µg/ml of ampicillin. The cell pellet was resuspended with 25 ml of buffer containing 50 mM Tris-HCl pH 8.0, 0.1 % TritonX100, 1 mM DTT and lysed by sonication. Proteins were precipated from the soluble crude extract by addition of 2 ml of 10 % polymin P pH 8.0 and centrifuged at 5000 g for 10 min. The polymin P pellet was resuspended with 25 ml of 50 mM Tris-HCl pH 8.0, 0.2 M NaCl, 1 mM DTT and centrifuged again. Solid ammonium sulfate (AS) was added to the supernatant to 35 % saturation. The AS precipitate was recovered by centrifugation at 20000 g for 20 min, and resuspended in 7 ml of buffer 50 mM Tris-HCl pH 8.0, 0.2 M NaCl, 1 mM DTT and 7.86 % AS weight/volume (15 % saturation). This solution was loaded onto a 5 ml Phenyl HP column (GE Healthcare) equilibrated with 50 mM Tris-HCl pH 8.0, 7.86 % AS. Proteins were eluted with an inverted gradient (7.86-0 % AS) in a 50 mM Tris-HCl pH 8.0 buffer and elution was monitored by SDS-PAGE. The PcrA-containing fractions were directly loaded on a Heparin HP column (GE Healthcare) equilibrated with 50 mM Tris-HCl pH 8.0, 100 mM NaCl, 1mM DTT. Proteins were eluted with a 50 ml gradient of 0.1 to 1 M NaCl. The peak fractions (around 0.5 M NaCl) were pooled and dialysed using 50 kDa cut-off dialysis tubing (Spectrapor) against 50 mM Tris-HCl pH 8.0, 200 mM NaCl, 1 mM DTT, 50 % glycerol. PcrA was obtained at 95 % purity and store at -20°C.

Protein concentrations were determined using Bradford reagent (Bio-Rad) as recommended by the supplier.

**Gel filtration assays**

High-resolution assays using Superdex 200 HR10/30 columns (GE) were carried out as described previously [5]; the buffer used was 50 mM Tris-HCl pH 8, 0.2 M NaCl, 1 mM DTT.

**Western blot analysis**

Whole protein extracts were prepared from *B. subtilis* cells grown to mid-log phase in LB supplemented with erythromycin, spectinomycin and IPTG, with or without D-Xylose. Cells were lysed in 20 mM Tris-HCl pH 8, 0.15 M NaCl, 10 mM EDTA pH 8 by addition of lysozyme (10 mg/ml) and incubation for 10 min at 0°C and 10 min at 37°C. After 3 freeze-thaw cycles, whole protein extracts were denatured in loading buffer, fractioned by 12% SDS-PAGE and transferred to nitrocellulose membrane (Protran, Whatman). SSB proteins were immunodetected with rabbit polyclonal anti-SSB raised against the SSBΔ66 protein purified from *E. coli*, using an ECL+ kit (GE Healthcare) with peroxidase-coupled goat anti-rabbit antibody (SIGMA, A6154). Western blots were analyzed using Multi-Gauge software (Fujifilm).

**References**

1. Lewis PJ, Marston AL (1999) GFP vectors for controlled expression and dual labelling of protein fusions in *Bacillus subtilis*. Gene 227: 101-110.

2. Lecointe F, Serena C, Velten M, Costes A, McGovern S, et al. (2007) Anticipating chromosomal replication fork arrest: SSB targets repair DNA helicases to active forks. Embo J 26: 4239-4251.

3. Hartl B, Wehrl W, Wiegert T, Homuth G, Schumann W (2001) Development of a new integration site within the *Bacillus subtilis* chromosome and construction of compatible expression cassettes. J Bacteriol 183: 2696-2699.

4. Dervyn E, Suski C, Daniel R, Bruand C, Chapuis J, et al. (2001) Two essential DNA polymerases at the bacterial replication fork. Science 294: 1716-1719.

5. Velten M, McGovern S, Marsin S, Ehrlich SD, Noirot P, et al. (2003) A two-protein strategy for the functional loading of a cellular replicative DNA helicase. Mol Cell 11: 1009-1020.
